# Supplementary material for: Protein Kinase C-β Dictates B Cell Fate by Regulating Mitochondrial Remodeling, Metabolic Reprogramming, and Heme Biosynthesis
Source: Immunity. 2018 Jun 19;48(6):1144–1159.e5. doi: 10.1016/j.immuni.2018.04.031 (PMC6015119; doi:10.1016/j.immuni.2018.04.031)
Supplement: Document S1. Figures S1–S6 [file mmc1.pdf]

Immunity, Volume *48*

Supplemental Information

Protein Kinase C-β Dictates B Cell Fate by Regulating Mitochondrial Remodeling, Metabolic Reprogramming, and Heme Biosynthesis

Carlson Tsui, Nuria Martinez-Martin, Mauro Gaya, Paula Maldonado, Miriam Llorian, Nathalie M. Legrave, Merja Rossi, James I. MacRae, Angus J. Cameron, Peter J. Parker, Michael Leitges, Andreas Bruckbauer, and Facundo D. Batista

Supplemental Information

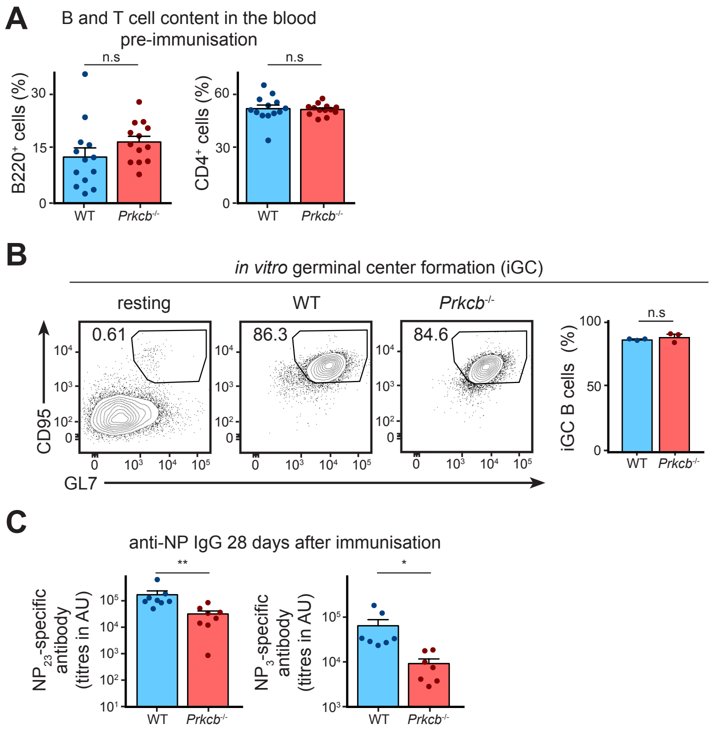

Figure S1. PKCβ depletion in the B cell compartment impairs T-dependent immune response. ***Related to Figure 1.***

(A) Quantifications showing percentages of B ($B220^+$) and $CD4^+$ T cells in the blood 8 weeks after bone marrow injection. Each dot represents one animal.

(B) Primary B cells from WT and *Prkcb*$^{-/-}$ mice cultured using iGC culture were analyzed using flow cytometry for the expression of GL-7 and CD95 after 4 days. Gated populations from three independent experiments were quantified.

(C) Sera were collected 28 days after NP-KLH immunization and anti-NP_{23} and anti-NP_3 IgG titres were measured using ELISA. All data are pooled from at least 2 independent experiments with at least 3 mice in each group. Error bars represent S.E.M.

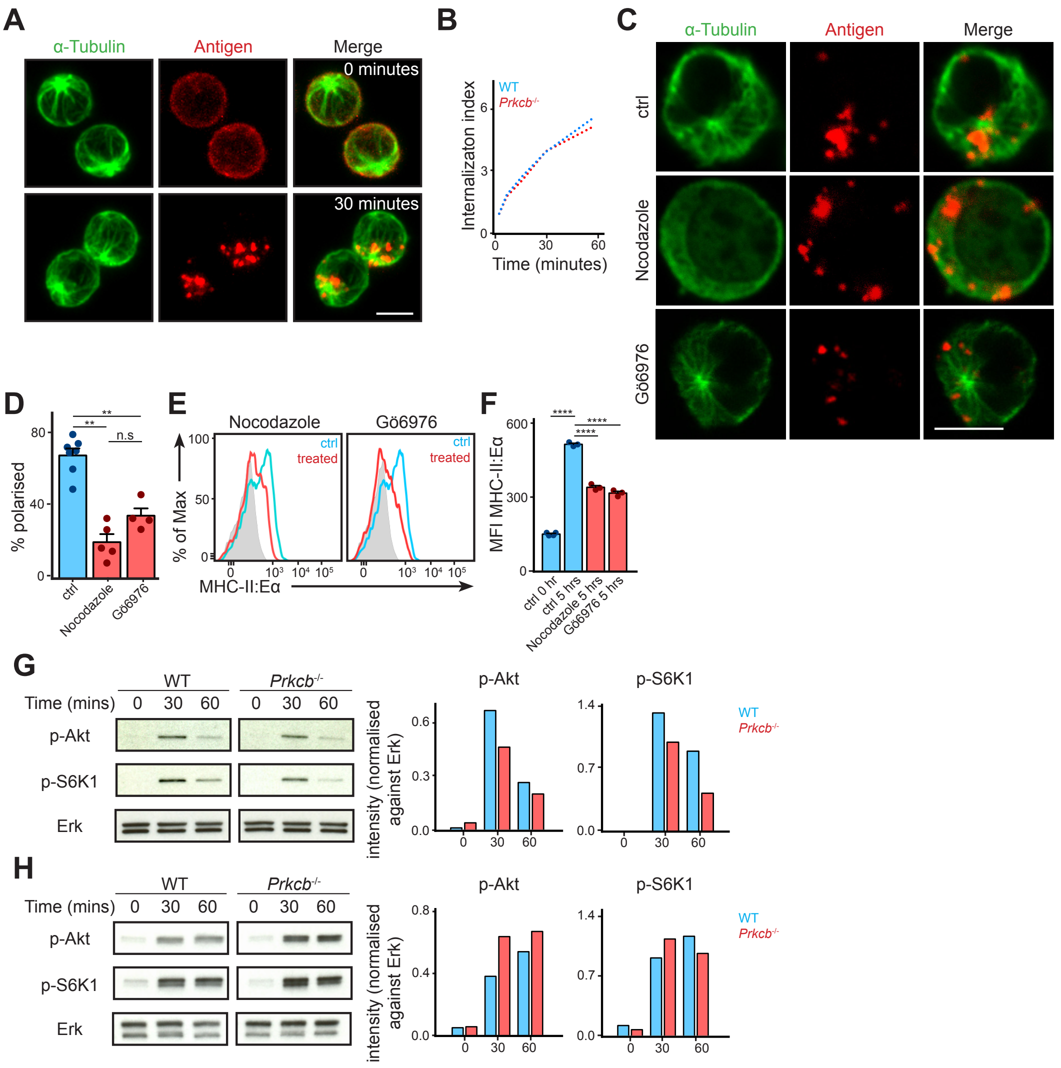

Figure S2. Antigen positioning is correlated with antigen presentation. ***Related to Figure 2.***

(A) Representative confocal images (63x objectives) showing primary B cells before and after stimulation with Alexa Fluoro 647-conjugated anti-IgM for 30 minutes. Bars, 5 µm. Data are representative of 2 independent experiments.

(B) Graph showing the internalization kinetics of antigen in WT and *Prkcb*$^{-/-}$ B cells stimulated (see *materials and* methods) for the indicated time points. Data are representative of 2 independent experiments.

(C) Representative confocal images (63x objectives) showing primary WT B cells stimulated with Alexa Fluoro 647-conjugated anti-IgM for 30 minutes in the absence and presence of microtubule depolymerizing agent nocodazole, PKC inhibitors Gö6976. Bars, 5 µm. Data are representative of 3 independent experiments.

(D) Samples were inspected using confocal microscopy and the proportions of cells with polarized antigen close to the MTOC were determined visually. Quantifications are pooled from 2 independent experiments with at least 40 cells per group and data analyzed using two-way ANOVA.

(E) Representative plots show the expression of surface MHC-II:Eα peptide after 5 hours detected by anti-MHC-II:Eα antibody of WT primary B cells that were incubated with microspheres in the presence of nocodazole or Gö6976. Data are representative of 2 independent experiments.

(F) MFI of MHC-II:Eα was quantified and analyzed using two-way ANOVA. Data are representative of 3 independent experiments.

(G and H) Western blots and the corresponding quantifications representing one experiment showing the induction of the PI3K pathway when B cells from WT and *Prkcb*$^{-/-}$ mice were given (G) coated-microspheres or (H) CpG and anti-IgM. Error bars represent S.E.M.

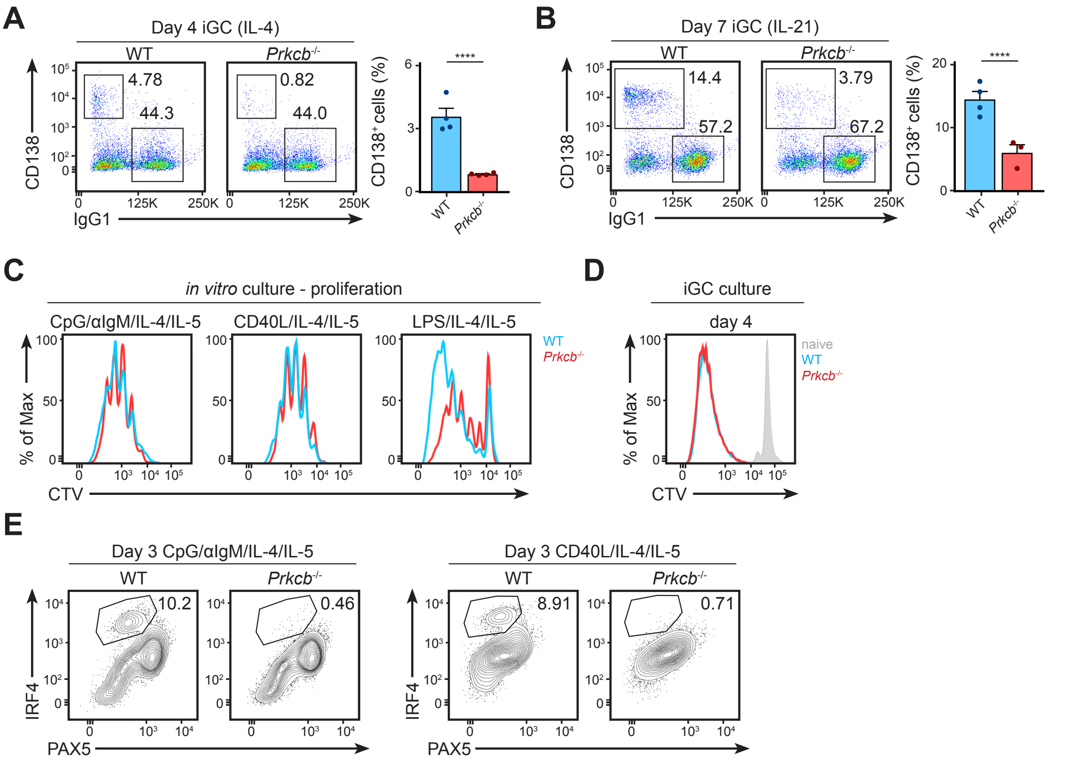

Figure S3. ***Prkcb*$^{-/-}$ B cells are intrinsically defective in plasma cell differentiation.** ***Related to Figure 3.***

(A-B) Primary B cells from WT and *Prkcb*$^{-/-}$ mice cultured using iGC culture were analyzed using flow cytometry for the expression of CD138 and IgG1 after (A) 4 days (IL-4 supplemented) and (B) 7 days (IL-4 and IL-21 supplemented) of culture. CD138^{hi} populations from three independent experiments were quantified.

(C) CTV-labelled primary B cells purified from WT and *Prkcb*$^{-/-}$ mice were cultured for 4 days in the presence of CpG and anti-IgM, CD40L or LPS (all in the presence of IL-4 and IL-5). Representative histograms of CTV dilutions are shown.

(D) CTV-labelled primary B cells from WT and *Prkcb*$^{-/-}$ mice cultured using iGC culture for 4 days. Representative histogram of CTV dilutions as analyzed using flow cytometry are shown.

(E) Primary B cells purified from WT and *Prkcb*$^{-/-}$ mice were cultured with CpG and anti-IgM or CD40L in the presence of IL-4 and IL-5 for 3 days. Contour plots of the expressions of PAX5 and IRF4 on day 3 of culture with the specified stimuli combinations. All data are representative from at least 3 independent experiments. Error bars represent S.E.M.

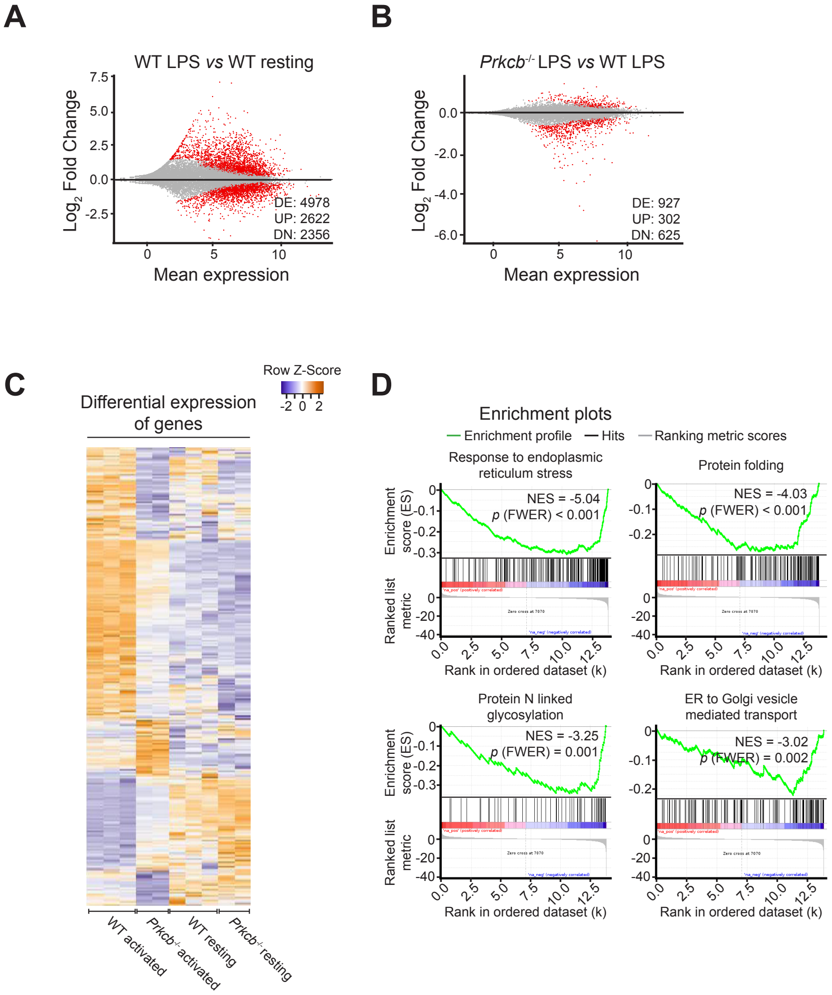

Figure S4. *Prkcb$^{-/-}$* B cells expressed distinct transcriptome after activation compared to WT cells. *Related to Figure 3 and 4.*

(A and B) MA plots ($\log_2$ Mean expression values on x-axis and $\log_2$ Fold change on y-axis) showing in red differentially expressed genes (*p*-adj < 0.05) in (A) activated WT B cells compared to resting WT B cells and (B) activated *Prkcb$^{-/-}$* B cells compared to activated WT B cells. DE: differentially expressed. UP: upregulated ($\log_2$ fold change > 0). DN: down-regulated ($\log_2$ fold change < 0).

(C) Hierarchical clustered heat map showing all 927 differentially expressed genes in B cells obtained from WT (n=3) and *Prkcb$^{-/-}$* (n=2) mice before and after LPS stimulation *in vitro*.

(D) GSEA enrichment plots showing significantly underrepresented gene sets (within the top 58 hits) in activated *Prkcb$^{-/-}$* B cells compared to activated WT cells.

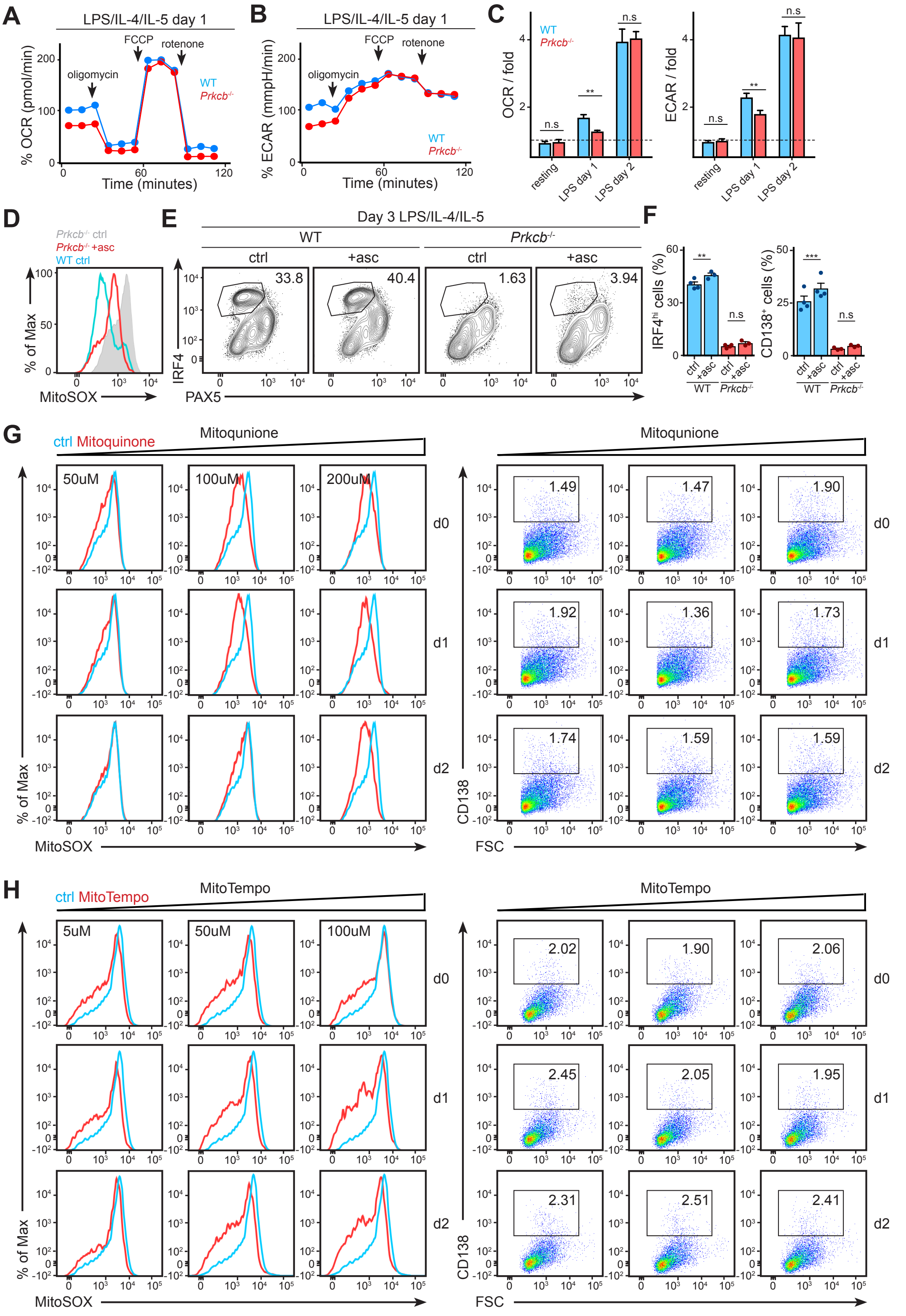

Figure S5. PKCβ functions as a regulator of mitochondrial homeostasis in activated B cells. ***Related to Figure 4.***

(A and B) Representative OCR (A) and ECAR (B) obtained from extracellular flux assay of WT and *Prkcb*$^{-/-}$ B cells stimulated with LPS, IL-4 and IL-5. Arrows indicate the time when each specified ETC modulators are added to the assay.

(C) Quantifications of oligomycin-sensitive OCR and steady state ECAR normalized to resting WT cells are shown. Data are pooled of at least 2 independent experiments with 2 mice in each group.

(D-F) Primary WT and *Prkcb*$^{-/-}$ B cells were cultured with LPS, IL-4 and IL-5 in the absence and presence of 200 µM ascorbic acid (added on day 1). (D) Accumulation of mROS and (E) contour plots of IRF4 and PAX5 expression. (F) Quantifications showing amount of IRF4^{hi} and CD138^{+} cells are shown in the same experimental setup. Data is representative of at least two independent experiments of two mice in each group.

(G and H) Primary WT and *Prkcb*$^{-/-}$ B cells were cultured with LPS, IL-4 and IL-5 in the absence and presence of (G) Mitoquinone or (H) MitoTempo. mROS accumulation and CD138 expression were analyzed using flow cytometry on day 3. Error bars represent S.E.M.

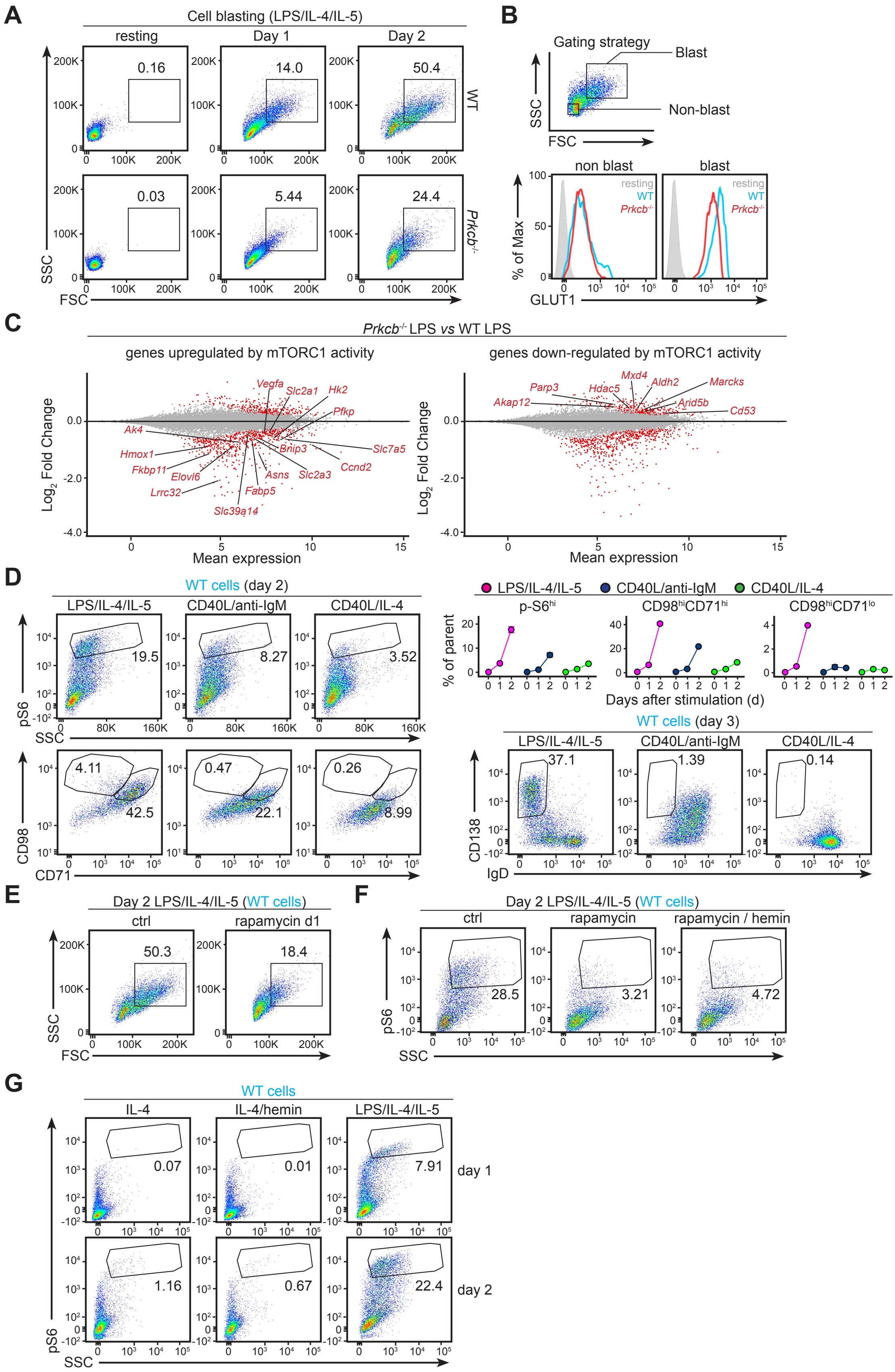

Figure S6. PKCβ regulates mTORC1 activity in activated B cells. ***Related to Figure 6.***

(A) Primary B cells purified from WT and *Prkcb*$^{-/-}$ mice were cultured with LPS, IL-4 and IL-5 for 2 days. The cell blasting kinetics was analyzed by flow cytometry on day 0, 1 and 2.

(B) B cells purified from WT and *Prkcb*$^{-/-}$ mice were cultured with LPS, IL-4 and IL-5 for 2 days. The cells were categorized into "blast" and "non-blast" using FSC. The total expression of GLUT1 within each population was measured using flow cytometry. GLUT1 expression in resting B cells is represented in grey.

(C) MA plots as in figure 3G. Highlighted in red are genes associated with (left) high mTORC1 activity and (right) low mTORC1 activity that are downregulated and upregulated, respectively, in activated *Prkcb*$^{-/-}$ cells compared to WT cells.

(D) Primary WT B cells were cultured in various stimuli as specified for 3 days. The mTORC1 activity (top left), CD98 and CD71 expression (bottom left) of the cells were monitored and quantitated (top right). The extent of plasma cell differentiation was measured on day 3 (bottom right).

(E) WT primary B cells were stimulated with LPS, IL-4 and IL-5 for 2 days. Rapamycin was added on day 1 of the culture and cell blasting was measured using flow cytometry on day 2.

(F) WT primary B cells were stimulated with LPS, IL-4 and IL-5 for 2 days. Rapamycin and hemin was added to the culture on day 1. Phosphorylation of S6 was measured using flow cytometry on day 2.

(G) WT primary B cells were cultured with IL-4 alone, IL-4 plus hemin and LPS, IL-4 and IL-5 for 2 days. Phosphorylation of S6 was analyzed using flow cytometry on day 1 and day 2. Error bars represent S.E.M.
