# Supplementary material for: Protein Kinase C-β Dictates B Cell Fate by Regulating Mitochondrial Remodeling, Metabolic Reprogramming, and Heme Biosynthesis
Source: Immunity. 2018 Jun 19;48(6):1144–1159.e5. doi: 10.1016/j.immuni.2018.04.031 (PMC6015119; doi:10.1016/j.immuni.2018.04.031)
Supplement: Document S2. Article plus Supplemental Information [file mmc3.pdf]

Immunity

Article

Protein Kinase C-β Dictates B Cell Fate by Regulating Mitochondrial Remodeling, Metabolic Reprogramming, and Heme Biosynthesis

Graphical Abstract

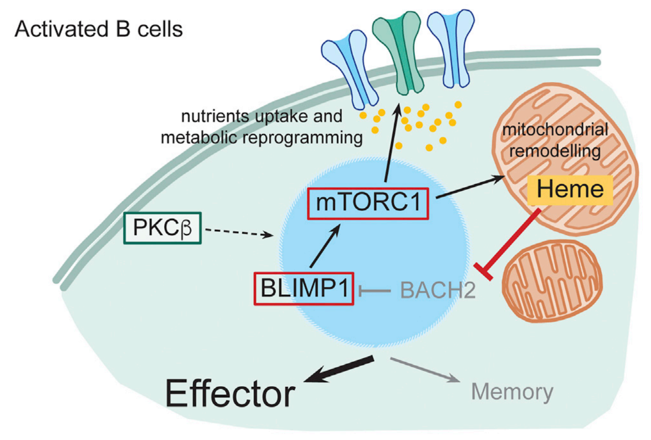

Highlights

- PKCβ in B cells promotes GC response and plasma cell differentiation *in vivo*
- PKCβ regulates antigen polarization and antigen presentation in B cells
- PKCβ drives mitochondrial remodeling and metabolic reprogramming in B cells
- Metabolic reprogramming couples heme accumulation to instruct effector cell fate

Authors

Carlson Tsui, Nuria Martinez-Martin, Mauro Gaya, ..., Michael Leitges, Andreas Bruckbauer, Facundo D. Batista

Correspondence

carlson.tsui@crick.ac.uk (C.T.), fbatista1@mgh.harvard.edu (F.D.B.)

In Brief

Lymphocyte activation is associated with major changes in metabolism. Tsui and colleagues demonstrate that PKCβ promotes metabolic reprogramming to drive effector fate decision in B cells.

Tsui et al., 2018, Immunity *48*, 1144–1159
June 19, 2018 © 2018 The Author(s). Published by Elsevier Inc.
https://doi.org/10.1016/j.immuni.2018.04.031

CellPress

Cell Press

Immunity
Article

Protein Kinase C-β Dictates B Cell Fate by Regulating Mitochondrial Remodeling, Metabolic Reprogramming, and Heme Biosynthesis

Carlson Tsui,[1,11,*] Nuria Martinez-Martin,[1,10,11] Mauro Gaya,[2] Paula Maldonado,[1] Miriam Llorian,[3] Nathalie M. Legrave,[4] Merja Rossi,[4] James I. MacRae,[4] Angus J. Cameron,[5] Peter J. Parker,[6,7] Michael Leitges,[8] Andreas Bruckbauer,[1,9] and Facundo D. Batista[1,2,12,*]

[1]Lymphocyte Interaction Laboratory, The Francis Crick Institute, London NW1 1AT, UK
[2]Ragon Institute of MGH, MIT and Harvard, Cambridge, MA 02139, USA
[3]Bioinformatics, The Francis Crick Institute, London NW1 1AT, UK
[4]Metabolomics, The Francis Crick Institute, London NW1 1AT, UK
[5]Barts Cancer Institute, Queen Mary University of London, London EC1M 6BQ, UK
[6]Protein phosphorylation Laboratory, The Francis Crick Institute, London NW1 1AT, UK
[7]School of Cancer and Pharmaceutical Sciences, King's College, London SE1 1UL, UK
[8]Biotechnology Centre of Oslo, University of Oslo, 0349 Oslo, Norway
[9]FILM, Imperial College London, London SW7 2BB, UK
[10]Present address: Centro de Biología Molecular Severo Ochoa, Universidad Autónoma de Madrid, Cantoblanco, 28049 Madrid, Spain
[11]These authors contributed equally
[12]Lead Contact
*Correspondence: carlson.tsui@crick.ac.uk (C.T.), fbatista1@mgh.harvard.edu (F.D.B.)
https://doi.org/10.1016/j.immuni.2018.04.031

SUMMARY

PKCβ-null (*Prkcb*$^{-/-}$) mice are severely immunodeficient. Here we show that mice whose B cells lack PKCβ failed to form germinal centers and plasma cells, which undermined affinity maturation and antibody production in response to immunization. Moreover, these mice failed to develop plasma cells in response to viral infection. At the cellular level, we have shown that *Prkcb*$^{-/-}$ B cells exhibited defective antigen polarization and mTORC1 signaling. While altered antigen polarization impaired antigen presentation and likely restricted the potential of GC development, defective mTORC1 signaling impaired metabolic reprogramming, mitochondrial remodeling, and heme biosynthesis in these cells, which altogether overwhelmingly opposed plasma cell differentiation. Taken together, our study reveals mechanistic insights into the function of PKCβ as a key regulator of B cell polarity and metabolic reprogramming that instructs B cell fate.

INTRODUCTION

B cells are key components of adaptive immunity that provide systemic defense against pathogenic infections through the production of highly specific antibodies. Antibody-secreting plasma cell generation depends on B cell activation, in which naive B cells are instructed to undergo cycles of proliferation and differentiation. The first signal required for B cell activation is initiated upon specific engagement of the B cell receptor (BCR) by its cognate antigen (Ag), resulting in a complex signaling cascade and uptake of the BCR:Ag complex. The internalized antigen is presented as cell surface peptides on class II major histocompatibility complex molecules (MHC-II) (Batista and Harwood, 2009), resulting in the engagement of specific CD4^{+} helper T cells and thus providing a second signal for maximal B cell activation. *In vivo*, activated B cells can rapidly differentiate to antibody-secreting plasma cells or enter into germinal centers (GCs) where BCR affinity maturation and class switch recombination take place (Rajewsky, 1996; McHeyzer-Williams and McHeyzer-Williams, 2005; De Silva and Klein, 2015; Victora and Nussenzweig, 2012). B cells in the GC shuttle between the light and the dark zone and BCRs with high antigen affinity are iteratively selected. These B cells exit the GC and differentiate into either high-affinity antibody secreting long-lived plasma cells or temporarily quiescent memory cells that can undergo plasma cell differentiation upon re-encountering the same antigen (McHeyzer-Williams and McHeyzer-Williams, 2005).

Previous efforts have established transcriptomic signatures that distinguish the changes of identity to predict B cell fate decision. For example, the high expression of the transcription factors BLIMP-1 (*Prdm1*) (Nutt et al., 2015; Shapiro-Shelef et al., 2003) and IRF4 (Klein et al., 2006; Sciammas et al., 2006) are reliable hallmarks of plasma cells (effector) development, while the expression of the transcription repressor BACH2 (Kometani et al., 2013; Shinnakasu et al., 2016) can predict memory progression. However, the signaling network and regulatory mechanisms required for B cell fate decisions are not fully understood. Recent data suggest that metabolic reprogramming during lymphocyte activation is, in part, important for regulating fate decisions in T cells (Pollizzi et al., 2016; Verbist et al., 2016; Yang et al., 2013).

Protein kinase Cs (PKCs) are signaling molecules that play key roles in many cellular processes. The PKC family is broadly

 © 2018 The Author(s). Published by Elsevier Inc.
This is an open access article under the CC BY license (http://creativecommons.org/licenses/by/4.0/).

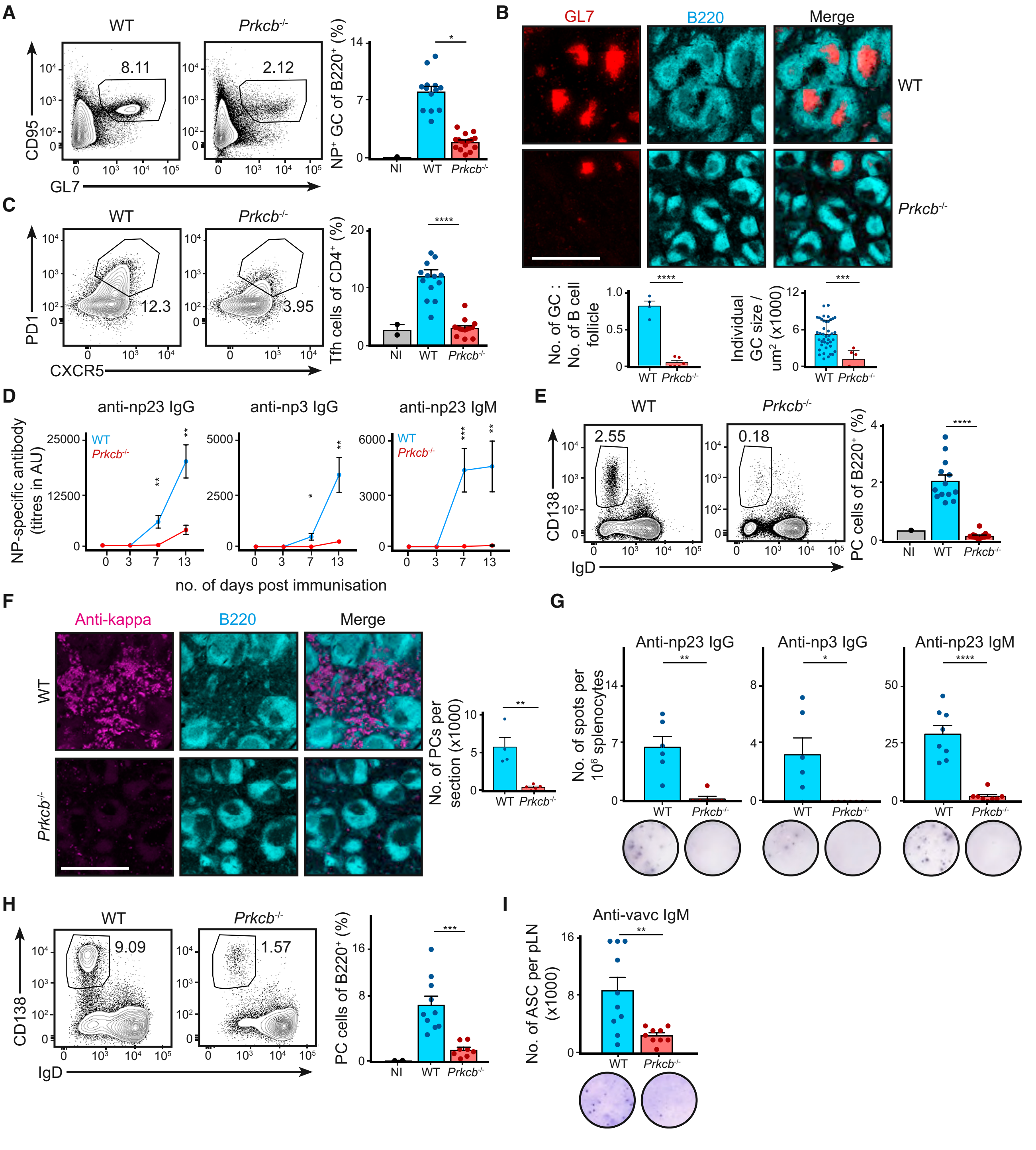

Figure 1. PKCβ Promotes Robust B Cell Response *In Vivo*

(A) Splenic GC B cell populations ($B220^+NP^+GL7^+CD95^+$) as analyzed by flow cytometry 13 days after NP-KLH and alum immunization of WT or *Prkcb*$^{-/-}$ chimeras.

(B) Confocal (20× objectives) tile images of the spleen of immunized WT and *Prkcb*$^{-/-}$ mice showing B220 and GL7 staining. Scale bar, 700 μm. The quantity and area of GCs were quantified using Imaris.

(C) Splenic Tfh cells ($CD4^+PD1^+CXCR5^+$) were quantified 13 days after immunization.

(D) NP-specific IgG and IgM titers on the specified days were determined by ELISA.

(E) Splenic plasma cells ($CD138^+IgD^-$) were quantified 13 days after immunization.

(F) Confocal (20× objectives) tile images of the spleen of immunized WT and *Prkcb*$^{-/-}$ mice showing surface B220 and intracellular κ staining. Scale bar, 700 μm. The amount of intracellular-κ^+ cells were quantified using Imaris.

(legend continued on next page)

divided into three subgroups: classical, novel, and atypical PKCs (Mellor and Parker, 1998). These subgroups differ in both protein sequences and mechanistic requirements for catalytic activity. The classical PKCs require cytosolic calcium (Ca^{2+}) for their activity, while the novel and atypical PKCs do not (Mellor and Parker, 1998). In B cells, PKCβ is the most highly expressed PKC member and plays a central part in propagating NF-κB signaling and cell proliferation downstream of the BCR (Saijo et al., 2002; Su et al., 2002). PKCβ-null mice exhibit impaired B cell development in the peritoneal cavity (Leitges et al., 1996) and (more mildly) the spleen (Leitges et al., 1996) and diminished humoral responses against T cell-dependent antigen (Leitges et al., 1996).

Here we reveal that PKCβ is essential for the regulation of antigen polarization and cell-fate decision in activated B cells. Using mice whose B cells lack PKCβ, we show that this protein is essential for GCs and plasma cell development upon immunization. Indeed, PKCβ-deficient B cells exhibit impaired antigen polarization and presentation that is likely to hinder GC development. Additionally, PKCβ promotes mTORC1-dependent mitochondrial remodeling and heme biosynthesis, resulting in BLIMP1-driven plasma cell differentiation (Watanabe-Matsui et al., 2011). Thus, our study provides mechanistic insights into the key role of PKCβ on B cell-fate decisions.

RESULTS

PKCβ Promotes Germinal Center Formation and Plasma Cell Differentiation

While *Prkcb*$^{-/-}$ mice exhibit severe immunodeficiency in response to T cell-dependent antigens (Leitges et al., 1996), it is unclear whether B cells contribute to the severe phenotype in these mice. To investigate whether the loss of PKCβ in B cells attenuates immune response *in vivo*, we initially generated mixed bone marrow (BM) chimeras whereby irradiated μMT mice (mature B cell-deficient) were reconstituted with a mixture of 80% μMT BM and either 20% WT (WT chimeras) or 20% *Prkcb*$^{-/-}$ (*Prkcb*$^{-/-}$ chimeras) BM. Thus, reconstituted *Prkcb*$^{-/-}$ chimeras would harbor *Prkcb*$^{-/-}$ B cells in an environment of mostly WT cells. The degree of reconstitution was assessed 6 to 8 weeks after adoptive transfer (Figure S1A). We then challenged WT and *Prkcb*$^{-/-}$ chimeras with NP_{23} conjugated to keyhole limpet haemocyanin (KLH) and Alum via intra-peritoneal injection and analyzed the immune response in the spleen at day 13 (Figures 1A–1G). In WT chimeras, we observed robust GC B cell development, indicated by the increased $B220^+GL7^+CD95^+$ population (Figure 1A). In contrast, we saw a severe reduction in the GC B cell population in *Prkcb*$^{-/-}$ chimeras (Figure 1A). By inspecting splenic sections using confocal microscopy, we observed that both the quantity and area of GC significantly decreased in immunized *Prkcb*$^{-/-}$ chimeras (Figures 1B). Our data suggest that PKCβ in B cells plays an important role in promoting GC reaction.

We asked whether this was an intrinsic feature in *Prkcb*$^{-/-}$ B cells and so we tested the ability of *Prkcb*$^{-/-}$ B cells to differentiate to GC-like B cells *in vitro*. We cultured primary WT and *Prkcb*$^{-/-}$ B cells with 40LB cells (Nojima et al., 2011) for 4 days and measured GL7 and CD95 expression using flow cytometry. We detected more than 80% of WT and *Prkcb*$^{-/-}$ B cells exhibiting GC-like features by day 4 (Figure S1B), suggesting that *Prkcb*$^{-/-}$ B cells were capable of forming GC B cells.

The formation of GCs *in vivo* depends on follicular T helper (Tfh) cells, as they provide essential co-stimulatory signals to B cells (Vinuesa and Cyster, 2011). We therefore compared the development of Tfh cells in WT and *Prkcb*$^{-/-}$ chimeras 13 days after immunization. We detected a robust induction of Tfh cells ($CD4^+CXCR5^+PD1^+$) in WT chimeras (Figure 1C). In contrast, Tfh cell development in immunized *Prkcb*$^{-/-}$ chimeras was reduced compared to immunized WT chimeras (Figure 1C), indicating that the absence of PKCβ in B cells affected the development of Tfh cells.

Robust GC reaction facilitates the production of class-switched antibodies and affinity maturation. In line with a defective GC response (Figures 1A and 1B), *Prkcb*$^{-/-}$ chimeras showed delayed and diminished appearance of NP_{23}-specific IgG titer as compared to WT chimeras (Figures 1D and S1C), suggesting that the production of class-switched antibodies was affected by the loss of PKCβ. Moreover, we found that the IgG derived from *Prkcb*$^{-/-}$ chimeras bound poorly to NP_3 (Figure 1D), reflecting a lack of high-affinity IgG in immunized *Prkcb*$^{-/-}$ chimeras. These data demonstrate that antibody class-switching and affinity maturation was affected in immunized *Prkcb*$^{-/-}$ chimeras.

Notably, we found that IgM production was completely abrogated in immunized *Prkcb*$^{-/-}$ chimeras (Figure 1D). As primary IgM titer is typically derived from extrafollicular plasma cells, which are independent of the GC, we asked whether plasma cell development was affected in the absence of PKCβ. We analyzed plasma cell differentiation in immunized WT and *Prkcb*$^{-/-}$ chimeras, and found that while WT chimeras exhibited a 5-fold increase of splenic plasma cells ($CD138^+IgD^-$) after immunization, no induction of plasma cell differentiation could be detected in the *Prkcb*$^{-/-}$ chimeras (Figure 1E). When inspecting splenic sections by confocal microscopy, we observed a marked reduction of plasma cells in the splenic extrafollicular area in *Prkcb*$^{-/-}$ chimeras versus WT chimeras (Figure 1F). These results were confirmed by ELISPOT analysis (Figure 1G). Taken together, our findings suggest that PKCβ plays an important role not only in promoting GC response but also in the generation of extrafollicular plasma cells.

To determine whether the loss of PKCβ in B cells would also affect plasma cell differentiation in the context of viral infection, we challenged WT and *Prkcb*$^{-/-}$ chimeras, intra-footpad, with 10^4 PFU of vaccinia virus and analyzed plasma cell differentiation in the draining popliteal lymph nodes (PLNs) 7 days after infection. We observed robust $CD138^+IgD^-$ plasma cells formation

(G) NP-specific IgG- and IgM-secreting cells 13 days after immunization were quantified by ELISPOT.
(H) Plasma cells ($CD138^+IgD^-$) in the PLN of WT or *Prkcb*$^{-/-}$ chimeras were quantified 7 days after vaccinia infection using flow cytometry.
(I) PLN VACV-specific IgM-secreting cells 7 days after infection were quantified using ELISPOT. All data are representative of at least 2 independent experiments. Each dot represents one mouse.
Error bars represent SEM. See also Figure S1.

in the PLNs of WT chimeras; in contrast, the development of these cells was reduced in the PLNs of infected *Prkcb*$^{-/-}$ chimeras (Figure 1H). Furthermore, ELISPOT analysis also revealed a reduction in the amount of VACV-specific IgM-secreting cells in the PLNs of infected *Prkcb*$^{-/-}$ chimeras compared to WT chimeras (Figure 1I). Together, these results suggest an important role for PKCβ in plasma cell differentiation *in vivo*.

Antigen Polarization in B Cells Requires PKCβ

Subsequently, we questioned how the loss of PKCβ affects B cell functions at the cellular level. BCR engagement results in a rapid internalization of the BCR:Ag complex and the polarization of antigen-containing compartments (Figure S2A), which was suggested to be dependent on classical PKCs (Siemasko et al., 1998). We therefore asked whether PKCβ is specifically required for antigen polarization in B cells. We stimulated primary WT, *Prkca*$^{-/-}$, and *Prkcb*$^{-/-}$ B cells for 30 min with Alexa647-conjugated anti-IgM and compared antigen polarization using confocal microscopy (Figures 2A and 2B). While about 60% of WT B cells and 50% of *Prkca*$^{-/-}$ B cells displayed polarized antigen (Figures 2A and 2B), it was reduced to 30% in *Prkcb*$^{-/-}$ B cells (Figure 2B). This result indicates that PKCβ is required for intracellular antigen polarization but not antigen internalization (see STAR Methods; Figure S2B).

Antigen polarization is thought to facilitate efficient antigen transfer to MHC-II molecules, required for the subsequent presentation to T cells (Siemasko et al., 1998); therefore, we examined whether *Prkcb*$^{-/-}$ B cells were less capable of antigen presentation (see STAR Methods). Accordingly, we stimulated primary WT, *Prkca*$^{-/-}$, and *Prkcb*$^{-/-}$ B cells with anti-IgM and Eα peptide-coated microspheres. The internalized Eα peptide is transferred to MHC-II (I-A^{b}) and presented on the cell surface (Rudensky et al., 1991), which we detected using an anti-MHC-II:Eα antibody (Figure 2C). In WT and *Prkca*$^{-/-}$ B cells, we observed robust Eα-presentation corresponding to a 3-fold increase in mean fluorescence intensity (MFI) by the end time point (Figures 2C and 2D). However, the MFI was decreased by 50% in *Prkcb*$^{-/-}$ B cells (Figures 2C and 2D), indicating that antigen presentation was impaired in *Prkcb*$^{-/-}$ but not *Prkca*$^{-/-}$ B cells. To assess how this might influence presentation to T cells, we assayed the response of WT and *Prkcb*$^{-/-}$ B cells in co-culture with OT-II CD4^{+} T cells (see STAR Methods). CellTrace Violet (CTV)-labeled WT and *Prkcb*$^{-/-}$ B cells were stimulated with anti-IgM and OVA-coated microspheres and co-cultured with CFSE-labeled OT-II T cells. After 3 days, we assessed the proliferation of B and T cells using flow cytometry. WT and *Prkcb*$^{-/-}$ B cells triggered comparable T cell proliferation; however, co-cultured *Prkcb*$^{-/-}$ B cells exhibited reduced proliferation compared to WT B cells (Figures 2E and 2F) despite having normal survival responses (Figures 2G and 2H). When *Prkcb*$^{-/-}$ B cells received CD40L, anti-IgM, and IL-4 (potent stimuli that mimicked B-T cell co-culture), they proliferated as robustly as WT cells (Figure 2I), indicating that the impaired proliferation of co-cultured *Prkcb*$^{-/-}$ B cells was likely a result of reduced T cell help. While *Prkcb*$^{-/-}$ B cells induced the proliferation of OT-II T cells (Li et al., 2001), the impaired antigen polarization in *Prkcb*$^{-/-}$ B cells correlated with a reduction in antigen presentation to T cells. To further affirm this, we analyzed the effects of nocodazole or Gö6976 (classical PKC inhibitor) on WT B cells. Treatment with either nocodazole or Gö8976 effectively blocked antigen polarization (Figures S2C and S2D) and Eα presentation (Figures S2E and S2F) compared to control. These findings demonstrate that the altered polarization of antigen-containing compartments impaired antigen presentation in *Prkcb*$^{-/-}$ B cells.

Antigen polarization is necessary to coordinate synergistic BCR and toll-like receptor 9 (TLR9) signaling in B cells (Chaturvedi et al., 2008; Eckl-Dorna and Batista, 2009). To test whether synergistic signaling was affected in *Prkcb*$^{-/-}$ B cells, CTV-labeled WT, *Prkca*$^{-/-}$, and *Prkcb*$^{-/-}$ B cells were cultured with anti-IgM and CpG-coated microspheres (see STAR Methods) and IL-4. We observed robust proliferation in both WT and *Prkca*$^{-/-}$ B cells after 3 days of stimulation, while *Prkcb*$^{-/-}$ B cells failed to proliferate to the same extent (Figure 2J). This difference was ablated when we stimulated *Prkcb*$^{-/-}$ B cells under conditions where antigen polarization is irrelevant, such as with unlinked CpG and anti-IgM (with IL-4) (Figure 2K; Chaturvedi et al., 2008). This suggests that synergistic signaling in *Prkcb*$^{-/-}$ B cells was attenuated due to the lack of antigen polarization. In line with this, *Prkcb*$^{-/-}$ B cells exhibited impaired PI3K signaling when stimulated with microspheres, but not unlinked CpG and anti-IgM (Figures S2G and S2H). Taken together, our data suggest that PKCβ is required for antigen polarization and presentation, which provides an explanation, at least in part, for the reduction in Tfh and GC B cells in *Prkcb*$^{-/-}$ chimeras.

PKCβ Instructs Plasma Cell Differentiation in B Cells

Although the role of PKCβ in antigen polarization likely contributes to the immunodeficiency in *Prkcb*$^{-/-}$ chimeras, the abrogation of plasma cell differentiation in *Prkcb*$^{-/-}$ chimeras made us wonder whether PKCβ plays other roles in B cell differentiation. To test this, we analyzed plasma cell differentiation of WT and *Prkcb*$^{-/-}$ B cells cultured *in vitro* with CpG, anti-IgM, IL-4, and IL-5 using flow cytometry. We found that plasma cell differentiation was reduced by more than 60% in stimulated *Prkcb*$^{-/-}$ B cells compared to those of the WT, while class-switch recombination (as measured by expression of IgG1) was unaffected (Figure 3A). We observed similar trends when *Prkcb*$^{-/-}$ B cells were cultured in CD40L (Figure 3B), LPS (Figure 3C), or with 40LB cells (Figures S3A and S3B), suggesting that this was independent of specific exogenous signals. Furthermore, we found that more *Prkcb*$^{-/-}$ cells underwent class switching compared to the WT in response to LPS and 40LB stimulation (Figures 3C, S3A, and S3B). Notably, these changes in differentiation did not correlate to cell proliferation (Figures S3C and S3D), suggesting that this might be a misstep in fate decision. Taken together, our data demonstrate that PKCβ plays an intrinsic and crucial part in promoting plasma cell differentiation in B cells.

The initiation of the plasma cell differentiation requires the dual-regulation of transcription factors, PAX5 and IRF4 (Nutt et al., 2015). To understand how PKCβ regulates plasma cell development, we analyzed IRF4 and PAX5 expression in LPS-stimulated (with IL-4 and IL-5) WT and *Prkcb*$^{-/-}$ B cells by flow cytometry (Figures 3D and 3E). We found that PAX5 and IRF4 expression increased in WT B cells on day 1 and 2 of the assay. On day 3, IRF4^{hi} cells downregulated PAX5 and adopted the typical plasma cell (IRF4^{hi}PAX5^{lo}) signature, with a doubling of this population detected by day 4 (Figures 3D and 3E). We

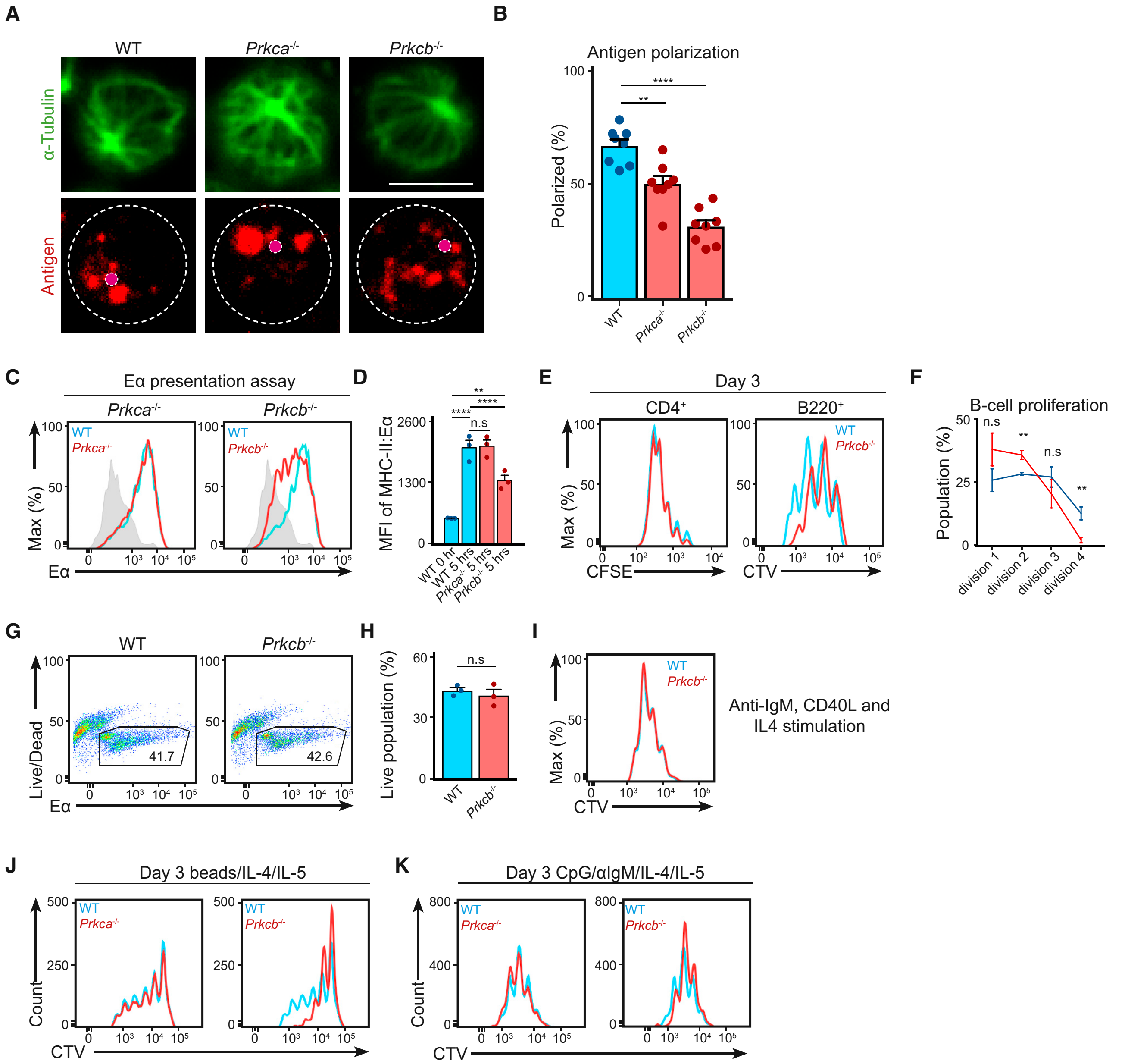

Figure 2. PKCβ Facilitates Intracellular Antigen Trafficking in B Cells

(A) Confocal images (63× objectives) of primary WT, *Prkca*$^{-/-}$, and *Prkcb*$^{-/-}$ B cells stimulated with Alexa647-conjugated anti-IgM for 30 min. Magenta dots indicate the corresponding MTOC. Bars, 5 μm.

(B) The extent of antigen polarization was quantified using ImageJ. Data were analyzed using two-way ANOVA.

(C) Representative plots of surface MHC-II:Eα expression as detected by anti-MHC-II:Eα antibody. Data are representative of at least 2 independent experiments.

(D) MFI of MHC-II:Eα was quantified. Data were analyzed using two-way ANOVA and are representative of 3 independent experiments.

(E) Primary WT and *Prkcb*$^{-/-}$ B cells incubated with anti-IgM and OVA-coated beads and co-cultured with OT-II T cells. Representative plots showing CFSE and CTV dilutions on day 3. Data are representative of at least 2 independent experiments.

(F) Quantification of CTV-divisions of WT and *Prkcb*$^{-/-}$ B cells in the co-culture experiment.

(G and H) Representative plots and quantification of cell recovery of WT and *Prkcb*$^{-/-}$ B cells after 3 days of co-culture.

(I) Representative plots of CTV dilutions of WT and *Prkcb*$^{-/-}$ B cells stimulated with CD40L, anti-IgM, and IL-4 for 3 days.

(J and K) Representative plots of CTV dilutions of WT, *Prkca*$^{-/-}$, and *Prkcb*$^{-/-}$ B cells stimulated with (J) anti-IgM and CpG-coated microspheres with IL-4 and IL-5, or (K) anti-IgM, CpG, IL-4, and IL-5 for 3 days. Data are representative of at least 2 independent experiments.

Error bars represent SEM. See also Figure S2.

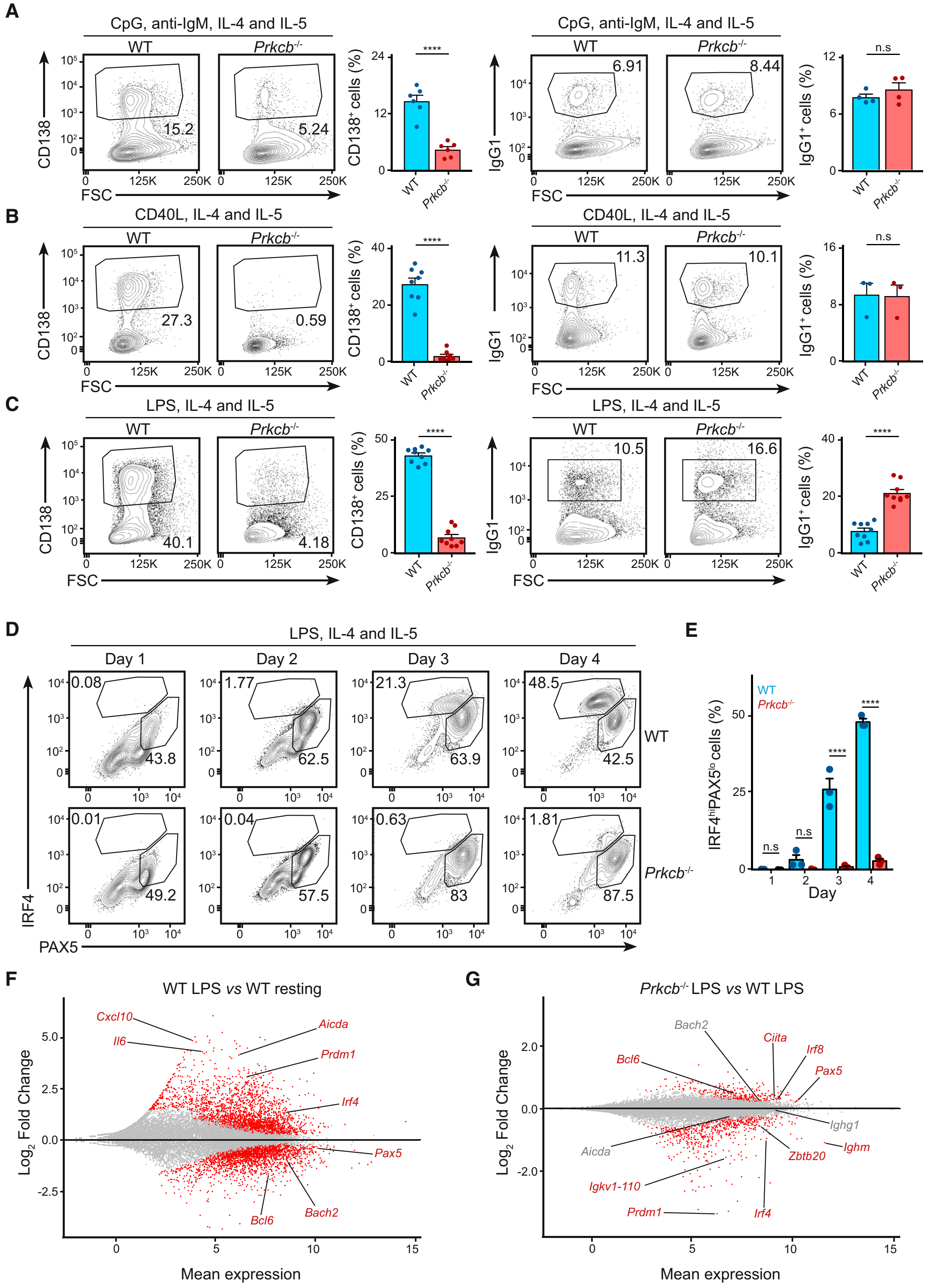

Figure 3. *Prkcb*$^{-/-}$ B Cells Fail to Commit to Plasma Cell Differentiation

(A–C) Representative plots and the corresponding quantifications of plasma cells and IgG1$^+$ cells of WT and *Prkcb*$^{-/-}$ B cells cultured in the presence of (A) CpG and anti-IgM, (B) CD40L, and (C) LPS (all in the presence of IL-4 and IL-5) for 4 days. Data are representative of at least 2 independent experiments with 2 mice in each group.

(legend continued on next page)

discerned few differences in IRF4 and PAX5 expression in activated *Prkcb*$^{-/-}$ B cells in the first 2 days of the assay; however, on day 3 and 4, despite maintaining an intermediate IRF4 expression, *Prkcb*$^{-/-}$ B cells did not acquire the IRF4^{hi}PAX5^{lo} signature (Figures 3D and 3E). Similar trends were observed using other combinations of stimuli (Figure S3E). Thus, these results reveal that *Prkcb*$^{-/-}$ B cells fail to surpass the IRF4 expression threshold that is required for plasma cell differentiation (Klein et al., 2006; Sciammas et al., 2006).

To elaborate on these findings, we performed genome-wide RNA sequencing (RNA-seq) on RNA extracted from WT and *Prkcb*$^{-/-}$ cells cultured with LPS, IL-4, and IL-5 for 2 days (see STAR Methods) (Figures S4A–S4C; Table S1). Consistent with the initiation of plasma cell differentiation (Nutt et al., 2015), we observed upregulation (UP, log_2 fold change > 0) of *Prdm1* and *Irf4* and downregulation (DN, log_2 fold change < 0) of *Bach2*, *Pax5*, and *Bcl6* in activated WT B cells compared to control cells (IL-4 stimulated) (Figures 3F and S4A). When we compared activated *Prkcb*$^{-/-}$ B cells with activated WT B cells (Figures S4B and S4C), we found that in contrast to WT cells, *Prkcb*$^{-/-}$ cells exhibited decreased *Prdm1*, *Irf4*, and *Ighm* (μ chain) expression (Figure 3G) and increased *Bcl6* and *Pax5* expression (Figure 3G). In line with unimpaired class switch recombination (Figures 3A–3C, S3A, and S3B), *Aicda*, *Bach2*, and *Ighg1* (γ1 chain) expression were normal in *Prkcb*$^{-/-}$ cells (Figure 3G; Muramatsu et al., 2000; Muto et al., 2004). Taken together, our data suggest that PKCβ promotes the transcriptomic program necessary for plasma cell differentiation.

PKCβ Facilitates Metabolic Reprogramming and Mitochondrial Remodeling in Activated B Cells

In order to understand how PKCβ instructs the plasma cell differentiation program, we applied gene set enrichment analysis (GSEA) to identify gross transcriptomic changes in activated *Prkcb*$^{-/-}$ B cells. Within the 58 most downregulated (FDR $\leq$ 0.001) gene sets in *Prkcb*$^{-/-}$ B cells, we noticed a predominant presence of gene sets relating to metabolism (Figure 4A). GSEA also identified gene sets relating to endoplasmic reticulum (ER) stress, protein modification, and anterograde membrane trafficking (Figure S4D), which are expected events preceding plasma cell differentiation. Specifically, expression of multiple mitochondrial-related genes such as *Atad1*, *Gpt2*, and *Hacd3* increased by 1.5-fold (log_2 fold change $\geq$ 0.5) after activation in WT cells but not in *Prkcb*$^{-/-}$ B cells (Figure 4B). Furthermore, mirroring the GSEA results, the expression of several well-characterized nutrient carriers such as *Slc2a1* (GLUT1) and *Slc3a2* (CD98) were also reduced in activated *Prkcb*$^{-/-}$ B cells (Figure 4C; Table S1).

To examine the metabolic fitness of these cells, we compared the metabolomic fingerprints of cultured WT and *Prkcb*$^{-/-}$ B cells (see STAR Methods). Notably, we observed reduced lactate abundance in the culture medium of *Prkcb*$^{-/-}$ cells compared to WT cells (Figure 4D). Next, we compared mitochondrial oxygen consumption rate (OCR) and extracellular acidification rate (ECAR, which reflects lactate production) in resting and activated WT and *Prkcb*$^{-/-}$ B cells using the extracellular flux (XF) system (Figures S5A–S5C). Accordingly, while *Prkcb*$^{-/-}$ B cells showed normal respiration under resting conditions, in line with their metabolomics fingerprints, they exhibited reduced basal OCR and ECAR after 1 day of culture in LPS compared to WT cells (Figures S5A–S5C). Comparable OCRs and ECAR were observed by day 2 (Figure S5C). These results were corroborated by ^{13}C-glucose labeling metabolomics on activated WT and *Prkcb*$^{-/-}$ B cells (see STAR Methods), wherein activated *Prkcb*$^{-/-}$ B cells showed decreased labeling in lactate and alanine and increased labeling in late TCA metabolites, compared to WT cells (Figure 4E). Collectively, these results suggest that PKCβ promotes metabolic reprogramming during B cell activation.

Given that the observed transcriptomic and metabolic changes likely involve the mitochondria, we investigated the mitochondrial status in WT and *Prkcb*$^{-/-}$ B cells before and after stimulation with LPS, IL-4, and IL-5. To this end, we combined MitoTracker green and MitoTracker red CMXROS staining to monitor fluctuations in mitochondrial mass and mitochondrial membrane potential (MMP) using flow cytometry (Figures 4F–4J). In WT B cells, LPS stimulation induced a rapid increase of mitochondrial mass and MMP, followed by a gradual decrease until the end of the assay on day 4 (Figures 4F and 4G). In contrast, while exhibiting normal mitochondrial mass and MMP at resting condition (Figures 4F and 4G), *Prkcb*$^{-/-}$ B cells showed increased accumulation of mitochondrial mass and MMP compared to WT cells on day 1 and 2 after activation (Figures 4F and 4G). Furthermore, most of the *Prkcb*$^{-/-}$ B cells failed to downregulate mitochondrial mass and MMP toward the end of the assay (Figures 4F–4I), in agreement with lack of plasma cell differentiation (Jang et al., 2015; Martínez-Martín et al., 2017). Likewise, the ratio between mitochondrial mass and MMP, which is indicative of mitochondrial quality, was reduced in activated *Prkcb*$^{-/-}$ B cells (Figure 4J), indicating that mitochondrial homeostasis was altered in activated *Prkcb*$^{-/-}$ B cells.

Mitochondria influence B cell fate via mROS generation (Jang et al., 2015). Importantly, while we observed similar mROS accumulation in resting WT and *Prkcb*$^{-/-}$ B cells, mROS accumulation was increased in *Prkcb*$^{-/-}$ B cells immediately following activation (since day 1) compared to WT B cells (Figure 4K), temporally coinciding with the relative mitochondrial quality (Figure 4J). Unlike WT cells, the addition of ascorbic acid (ROS scavenger) did not increase plasma cell differentiation in *Prkcb*$^{-/-}$ B cells (Figures S5D–S5F; Jang et al., 2015). Likewise, other mitochondrial-targeting antioxidants mitoquinone (MitoQ) and MitoTempo were ineffective in this context (Figures S5G and S5H), indicating that although high mROS might have suppressed plasma cell differentiation in *Prkcb*$^{-/-}$ B cells, low mROS alone was not sufficient to promote plasma cell

(D) Expressions of PAX5 and IRF4 in primary WT and *Prkcb*$^{-/-}$ B cells cultured in LPS, IL-4, and IL-5 for 4 days.
(E) The size of IRF4^{hi} cells (gated) was quantified. Data are of at least 2 independent experiments with 2 mice in each group.
(F and G) MA plot of genes that are differentially expressed (red) with p-adj < 0.05 in activated WT cells (F) or in activated *Prkcb*$^{-/-}$ cells (G) compared to resting WT cells.
Error bars represent SEM. See also Figure S3.

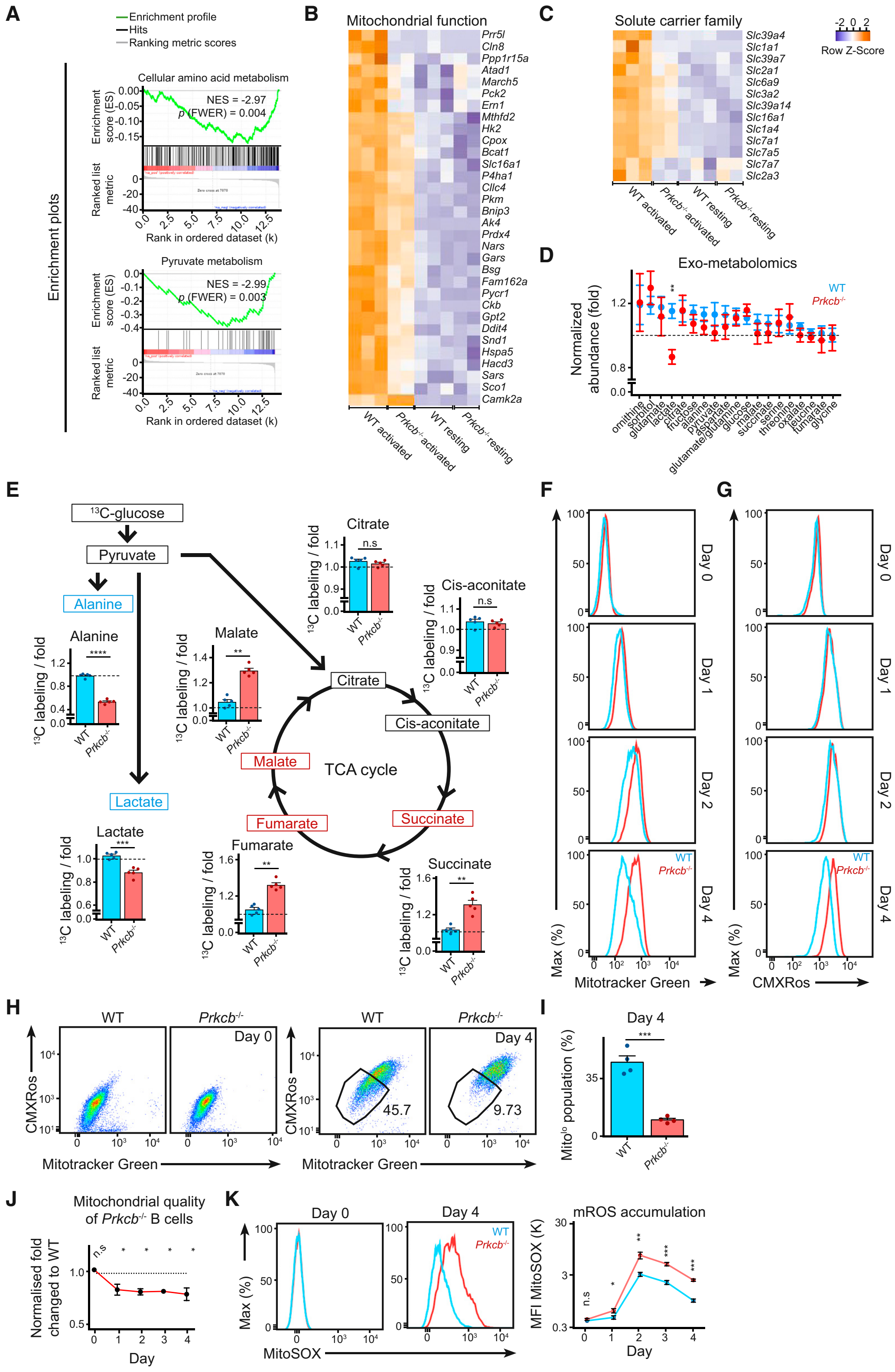

Figure 4. Activated *Prkcb*$^{-/-}$ B Cells Exhibited Abnormal Metabolism and mROS Accumulation

(A) GSEA plots of selected downregulated gene sets in *Prkcb*$^{-/-}$ B cells.

(B and C) Hierarchical clustered heatmaps showing expression of genes relating to (B) mitochondria and (C) solute carriers (*Slc*).

(legend continued on next page)

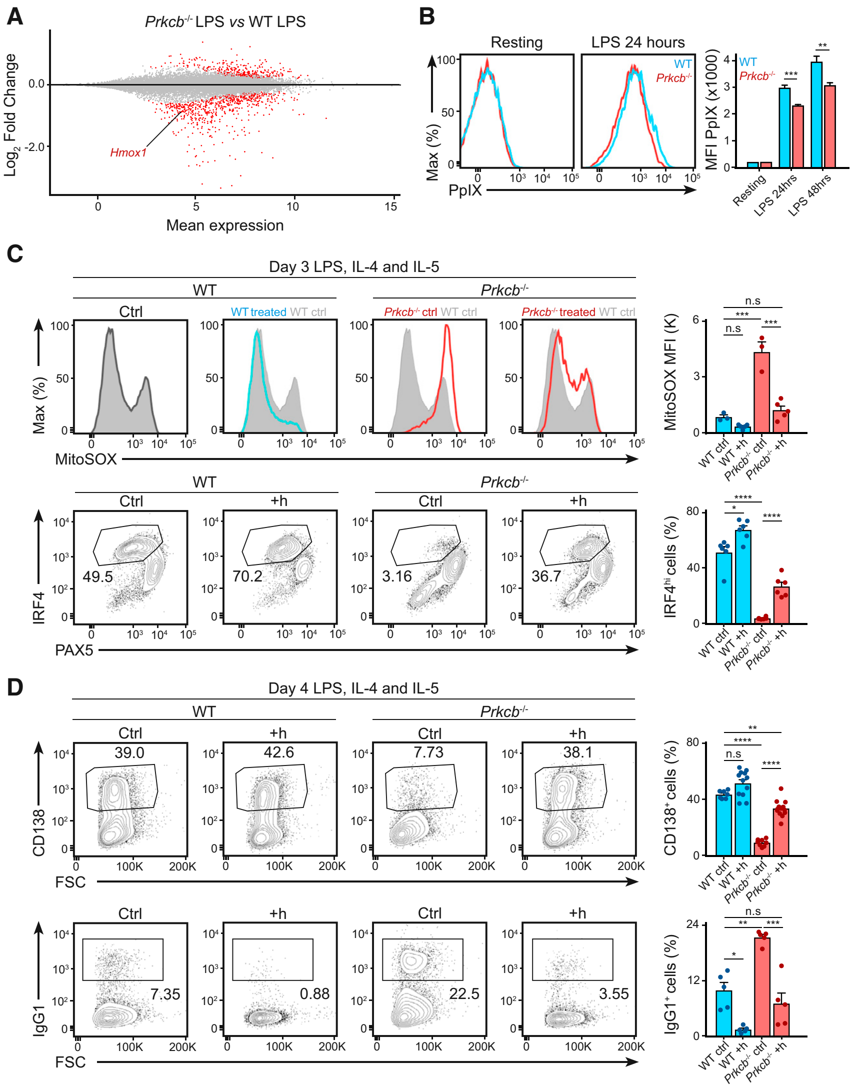

Figure 5. Heme Addition Restores Effector Differentiation in *Prkcb*$^{-/-}$ B Cells

(A) MA plot showing downregulation of *Hmox1* in *Prkcb*$^{-/-}$ B cells.

(B) The relative accumulation of PpIX as quantified by flow cytometry in WT and *Prkcb*$^{-/-}$ B cells cultured in LPS, IL-4, and IL-5.

(C and D) mROS, PAX5, and IRF4 expression, CD138^{+} and IgG1^{+} cells as quantified by flow cytometry in WT and *Prkcb*$^{-/-}$ B cells cultured in LPS, IL-4, and IL-5 for 4 days in the absence or presence of hemin (added on day 1). Data were analyzed using two-way ANOVA and are representative of at least 3 independent experiments.

Error bars represent SEM.

differentiation in the absence of PKCβ. Overall, our findings demonstrate that PKCβ is essential to promote the fitness of mitochondrial and non-mitochondrial metabolism and regulate mitochondrial status and mROS homeostasis.

Mitochondrial Metabolism Couples Heme Biosynthesis to Drive Plasma Cell Differentiation

We next questioned how metabolism could affect plasma cell differentiation. Plasma cell differentiation is associated with increased biosynthesis of heme (Watanabe-Matsui et al., 2011; Jang et al., 2015), a porphyrin that is capable of inhibiting the activity of the transcription factor BACH2 (Watanabe-Matsui et al., 2011). Heme biosynthesis is a metabolite-demanding multistep process (Ajioka et al., 2006) and is inhibited by high mROS (Jang et al., 2015). Given that *Prkcb*$^{-/-}$ B cells exhibited altered metabolism and mROS accumulation, we tested whether heme biosynthesis was impaired in *Prkcb*$^{-/-}$ B cells. We found that the expression of *Hmox1* (hemoxygenase-1), typically induced by heme accumulation (Watanabe-Matsui et al., 2011), was among the DN genes found in activated *Prkcb*$^{-/-}$ B cells (Figure 5A). We also observed that Protoporphyrin IX (PpIX, the final substrate of heme biosynthesis) accumulation was reduced in activated *Prkcb*$^{-/-}$ B cells compared to WT cells (Figure 5B), indicating that metabolic changes in activated *Prkcb*$^{-/-}$ B cells likely impaired heme biosynthesis and caused BACH2 hyperactivity.

Thus, to test whether heme supplementation would reverse fate decision in activated *Prkcb*$^{-/-}$ B cells, we cultured WT and *Prkcb*$^{-/-}$ B cells in LPS (with IL-4 and IL-5) in the presence or absence of exogenous hemin. On day 3 and 4, we analyzed mROS accumulation, IRF4 expression, plasma cell differentiation, and class switch recombination using flow cytometry (Figures 5C and 5D). Hemin addition reduced mROS accumulation and restored IRF4 expression (Figure 5C) and plasma cell differentiation (Figure 5D) in *Prkcb*$^{-/-}$ B cells. These changes corresponded with a reduction in class switch recombination (Figure 5D), indicating a hemin-driven fate-decision switch (Jang et al., 2015; Watanabe-Matsui et al., 2011). Thus, our data suggest that PKCβ promotes an activation-induced metabolic program necessary for mROS homeostasis and heme biosynthesis that is critical for B cell fate determination.

(D) The normalized abundance of polar metabolites in culture supernatant as determined using gas chromatography-mass spectrometry (GC-MS) of WT and *Prkcb*$^{-/-}$ B cells cultured in LPS, IL-4, and IL-5 for 2 days. Data are representative of 2 independent experiments of 2 mice in each group.

(E) Percentage of ^{13}C incorporation in activated WT and *Prkcb*$^{-/-}$ B cells. Each dot represents one mouse (n = 5) and is the average of 3 technical replicates.

(F–K) MFIs of MitoTracker Green (F), MitoTracker Red CMXRos (G), two-dimension representation (H and I), their ratios (J), and MFI of MitoSOX (K) in WT and *Prkcb*$^{-/-}$ B cells cultured in LPS, IL-4, and IL-5 for 4 days. Data are representative of 3 independent experiments.

Error bars represent SEM. See also Figures S4 and S5.

PKCβ Controls Mitochondrial Status and mROS Accumulation Partly through mTORC1

The mTORC1 signaling pathway is known to play an important role in cell growth, protein synthesis, and metabolism, as well as the regulation of mitochondrial biogenesis and function (Laplante and Sabatini, 2013; Morita et al., 2013). In order to examine whether the metabolic changes observed in *Prkcb*$^{-/-}$ B cells involved alterations in mTORC1 signaling, we compared mTORC1 activity in WT and *Prkcb*$^{-/-}$ cells before and after stimulation (Figures 6A, 6B, and S6A). Using flow cytometry, we noticed that a portion of WT B cells initiated mTORC1 signaling on day 1, characterized by ribosomal protein S6 phosphorylation (Figure 6A), cell blasting (Figure S6A), and surface expression of CD98 and CD71 (Figure 6B; Yang et al., 2013). This mTORC1-active population doubled by day 2 (Figures 6A, 6B, and S6A). In contrast, this population was reduced in activated *Prkcb*$^{-/-}$ B cells (Figures 6A, 6B, and S6A). Consistently, we observed decreased GLUT1 expression and altered expression of mTORC1-associated genes (Cunningham et al., 2007; Laplante and Sabatini, 2013; Yang et al., 2013) in activated *Prkcb*$^{-/-}$ B cells (Figures S6B and S6C). Importantly, while the upregulation of mTORC1 activity was a general feature of B cell activation (Figure S6D), high mTORC1 signaling and CD98 expression specifically correlated with plasma cell differentiation (Figure S6D). Taken together, these data provide evidence that PKCβ is important for mTORC1 signaling during early B cell activation.

Given that defects in mTORC1 signaling temporally coincides with alterations in metabolic reprogramming in *Prkcb*$^{-/-}$ cells, we questioned whether disruption of mTORC1 signaling in WT B cells was sufficient to affect cell fate. Accordingly, we cultured WT B cells in LPS (with IL-4 and IL-5) for 4 days and inhibited mTORC1 function using rapamycin at different stages of B cell activation. We analyzed mROS accumulation and plasma cell differentiation on day 3 and day 4 using flow cytometry (Figure 6C). Notably, we found that inhibition of mTORC1 signaling on day 1 enhanced mROS accumulation and reduced plasma cell differentiation, which was in line with our observations in *Prkcb*$^{-/-}$ B cells (Figure 6C). In contrast, we noticed that mTORC1 inhibition on either day 2 or 3 only mildly affected mROS accumulation and plasma cell differentiation (Figure 6C), suggesting that fate decision in B cells involves an early and somewhat transient wave of mTORC1 activity, corroborating recent findings (Ersching et al., 2017). Mechanistically, rapamycin treatment suppressed mTORC1 activity (Figure 6D), reduced cell size (Figure S6E), and decreased CD98 and CD71 expression (Figure 6E) and PpIX accumulation (Figure 6F), which was reminiscent of *Prkcb*$^{-/-}$ B cells. We thus questioned whether the lack of plasma cell differentiation rapamycin-treated WT cells was a consequence of reduced heme accumulation as in *Prkcb*$^{-/-}$ cells. To address this, we measured plasma cell differentiation of WT B cells in the presence of rapamycin alone or of rapamycin and hemin (Figures 6G and 6H). We found that hemin supplementation partially increased plasma cell differentiation in rapamycin-treated cells without alleviating mTORC1 inhibition (Figures 6G, 6H, and S6F), indicating that rapamycin inhibited plasma cell differentiation via heme homeostasis. Taken together, these results demonstrate that mTORC1 mediates plasma cell differentiation through mROS and heme homeostasis, providing a mechanism of the defects observed in *Prkcb*$^{-/-}$ B cells.

Early mTORC1 Activity Promotes and Sustains Effector Fate Commitment in B Cells

We were intrigued by the observation that hemin seemed to be more potent to *Prkcb*$^{-/-}$ cells than rapamycin-treated WT cells. To better understand the relationship between heme homeostasis, mTORC1, and plasma cell differentiation, we studied the effect of hemin and rapamycin on BLIMP1 expression in WT B cells using the *Prdm1*^{gfp} system (Figures 7A–7C; Kallies et al., 2004). We noticed that hemin not only induced BLIMP1 expression (Watanabe-Matsui et al., 2011) but also increased S6 phosphorylation, cell blasting, and CD98 expression (Figures 7A–7C). Importantly, resting B cells did not respond to hemin (Figure S6G), suggesting that while hemin promoted mTORC1 activity in activated B cells, it alone could not initiate mTORC1 signaling or plasma cell differentiation. On the other hand, rapamycin treatment not only inhibited mTORC1 activity, it also suppressed BLIMP1 expression, cell blasting, and CD98 expression (Figures 7A–7C). Collectively, our results strongly support a model of plasma cell differentiation that involves the crosstalk between mTORC1, BACH2, and BLIMP1 (Figure 7D).

To confirm that this was indeed the mechanism of rescue in *Prkcb*$^{-/-}$ B cells, we stimulated *Prkcb*$^{-/-}$ B cells *in vitro* with or without exogenous hemin and measured mTORC1 activity using flow cytometry (Figure 7E). Consistent with our hypothesis, we found that hemin addition increased S6 phosphorylation (Figure 7E) and CD98 expression (Figure 7E) in *Prkcb*$^{-/-}$ B cells, suggesting that hemin restored plasma cell differentiation via the indirect activation of mTORC1 in the absence of PKCβ. Collectively, our data had revealed a vital role of PKCβ in B cell fate decision through the initiation of the mTORC1-BACH2-BLIMP1 program.

We wanted to determine whether deregulation of the mTORC1-BACH2-BLIMP1 program underlies the lack of plasma cell differentiation in other systems that are known to have PI3K or mTOR signaling deficiencies, such as CD19-deficient B cells (Buhl et al., 1997; Keppler et al., 2015). We therefore cultured WT and *Cd19*$^{-/-}$ B cells in LPS (with IL-4 and IL-5) in the presence or absence of exogenous hemin (Figures 7F–7H). We found that while WT B cells exhibited high mTORC1 signaling and robust CD98 expression 2 days after culture (as shown previously), activated *Cd19*$^{-/-}$ B cells exhibited decreased mTORC1 activity and CD98 expression (Figure 7F), similar to *Prkcb*$^{-/-}$ B cells. Hemin supplementation led to increased CD98 expression (Figure 7G), increased IRF4 expression, plasma cell differentiation, and decreased class switch recombination in *Cd19*$^{-/-}$ B cells (Figure 7H). Taken together, our study proposes a model in which B cell metabolic reprogramming sustains the mTORC1-BACH2-BLIMP1 program and in turn drives effector differentiation in B cells.

DISCUSSION

We have uncovered the decisive role of PKCβ in B cell physiology: PKCβ mediates GC reaction and plasma cell development in response to T cell-dependent antigen challenge *in vivo*. While the immunodeficiency in *Prkcb*$^{-/-}$ chimeras appears to be a

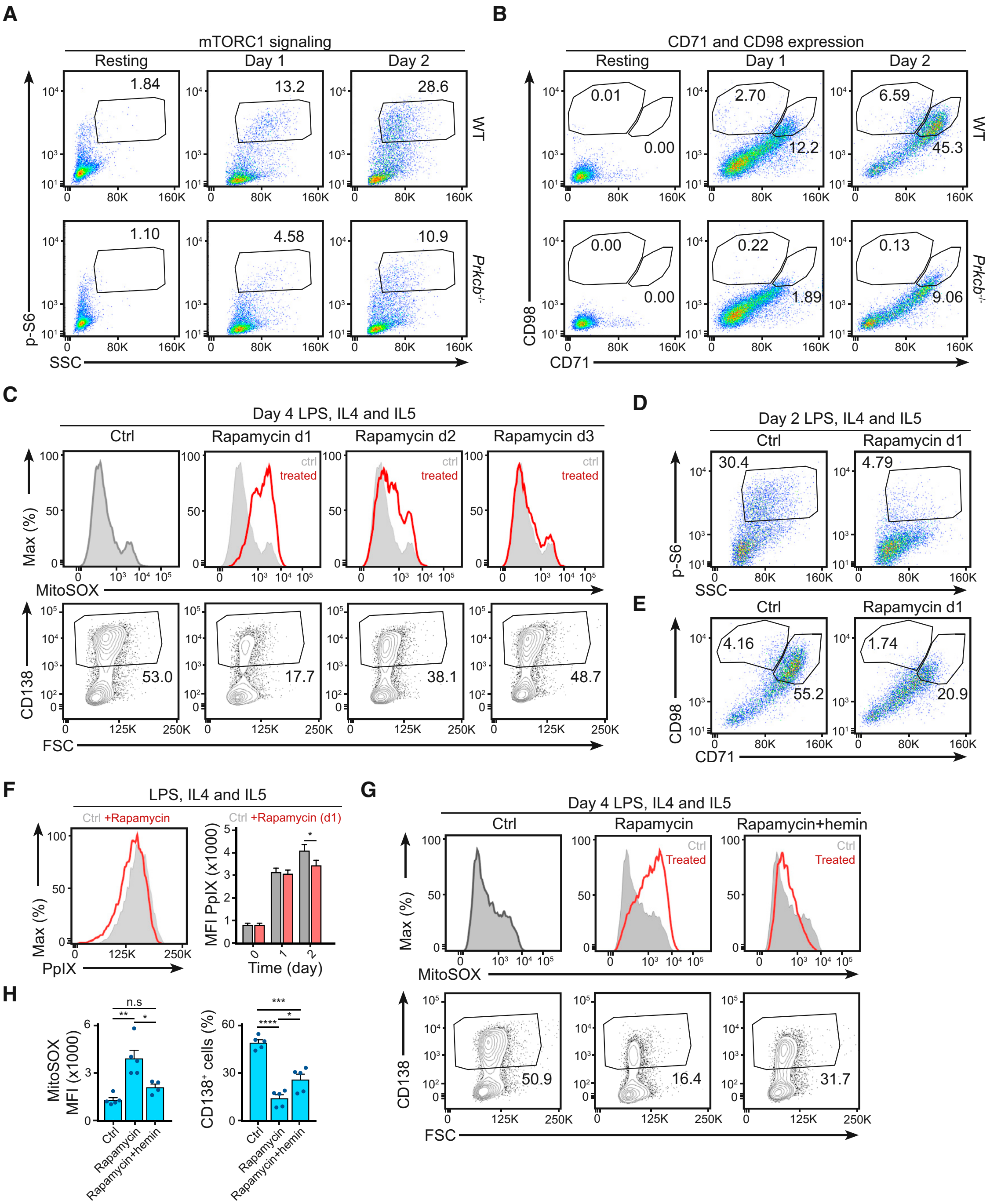

Figure 6. *Prkcb*$^{-/-}$ B Cells Had Impaired Early mTORC1 Signaling in Response to Activation

(A and B) Phosphorylation of S6, CD98, and CD71 surface expression as analyzed by flow cytometry of WT and *Prkcb*$^{-/-}$ B cells cultured in LPS, IL-4, and IL-5 for 2 days. Data are representative of at least 3 independent experiments.

(C) mROS accumulation and plasma cell differentiation as quantified by flow cytometry in WT B cells cultured in LPS, IL-4, and IL-5 for 4 days. Rapamycin was added at various time points as specified.

(D and E) S6 phosphorylation and CD98 and CD71 surface expression as analyzed using flow cytometry in WT B cells cultured in LPS, IL-4, and IL-5 for 2 days in the absence and presence of rapamycin (added on day 1).

(F) Relative abundance of PpIX as quantified by flow cytometry of WT B cells cultured in LPS, IL-4, and IL-5 for 2 days in the absence and presence of rapamycin (added on day 1). Quantification represents data from 3 independent experiments.

(G and H) mROS accumulation and plasma cell differentiation as quantified by flow cytometry of WT B cells cultured in LPS, IL-4, and IL-5 for 4 days in the presence of either rapamycin or rapamycin plus hemin (added on day 1). Data were analyzed using two-way ANOVA and are of 3 independent experiments. Error bars represent SEM. See also Figure S6.

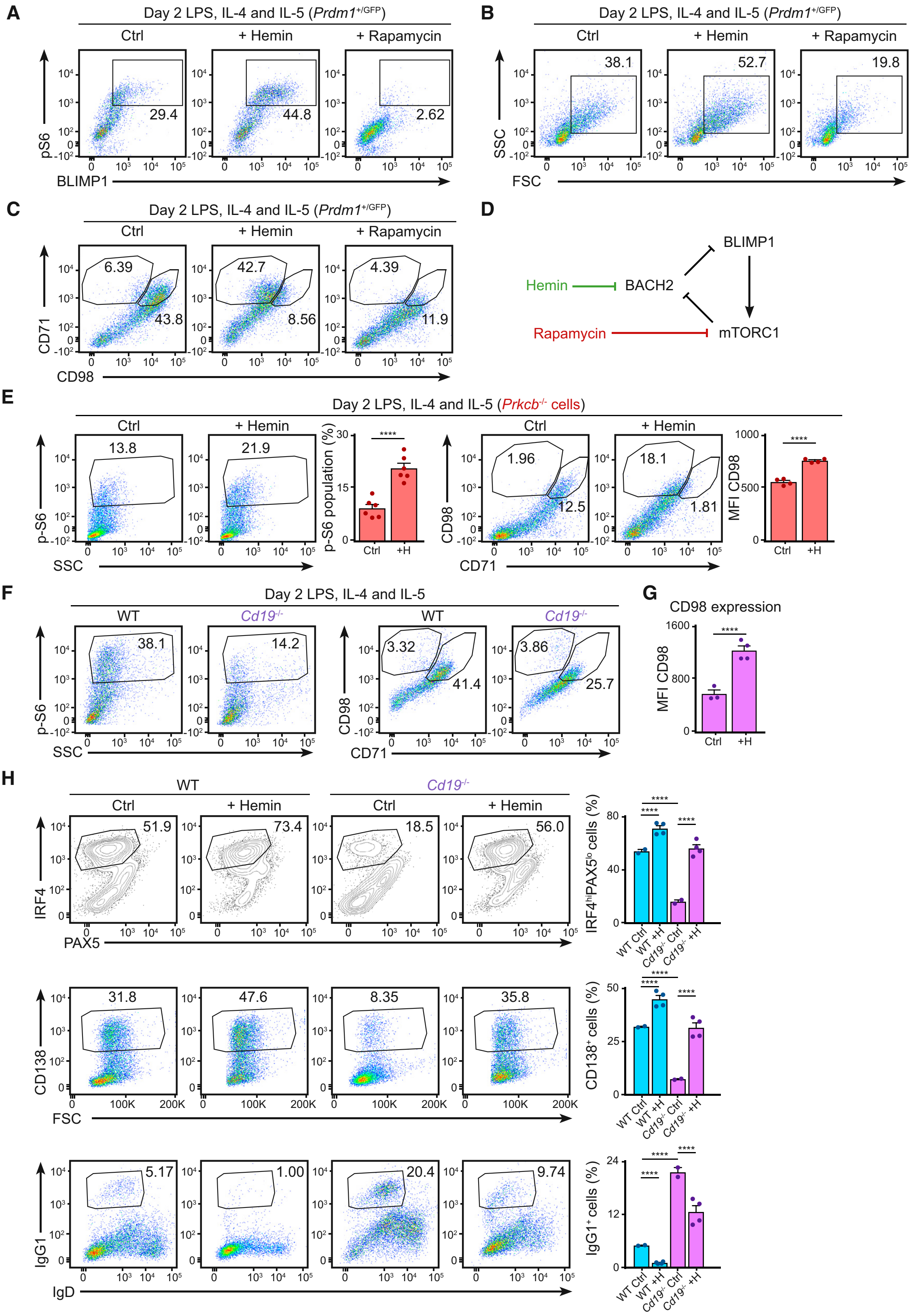

(legend on next page)

consequence of multiple B cell-intrinsic impairments, we reason that the loss of antigen polarization and presentation specifically contribute to GC defects. Indeed, previous study had revealed such correlation in Cdc42-deficient B cells (Burbage et al., 2015). Impaired antigen presentation leads to reduced T cell help *in vivo*, which could delay GC formation and affinity maturation, as observed in both *Prkcb*$^{-/-}$ and *Cdc42*$^{-/-}$ B cells. We speculate that the defects in antigen positioning in *Prkcb*$^{-/-}$ B cells likely affects antigen transfer to other secondary compartments such as TLR9^{+} vesicles (Chaturvedi et al., 2008), which may explain the poor synergistic signaling and proliferative response in these cells when stimulated with microspheres *in vitro*. At this stage, how PKCβ control antigen positioning is unclear, although we suspect that PKCβ regulates organelle trafficking (Siemasko et al., 1998; Kermorgant et al., 2003).

In line with our *in vivo* observations, PKCβ-null mice fail to elicit antibody titer upon primary T cell-dependent immunization (Leitges et al., 1996). However, this appears to be less severe in the recall response (Leitges et al., 1996), implying that PKCβ deficiency may not affect memory generation during the primary challenge. Consistent with this, activated *Prkcb*$^{-/-}$ cells were able to induce *Aicda*, *Bach2*, and *Ighg1* expression (Klein et al., 2006; Muramatsu et al., 2000; Muto et al., 2004), as well as undergoing class-switch recombination *in vitro*, which together support the notion that the development of class-switch compartment should be normal, if not enhanced in *Prkcb*$^{-/-}$ chimeras. Future works should address whether BCR signaling is affected in PKCβ-deficient memory B cells.

We have shown that the lack of an mTORC1-mediated metabolic reprogramming in *Prkcb*$^{-/-}$ B cells suppresses plasma cell differentiation. While mTORC1 inhibition is known to delay BACH2 downregulation and suppress plasma cells differentiation (Kometani et al., 2013), hyperactivity of mTORC1 enhances plasma cell differentiation (Benhamron et al., 2015). Here, we have shown that early mTORC1 signaling instructs plasma cell differentiation through the crosstalk to BACH2, BLIMP1, and metabolic reprogramming. We demonstrate that mTORC1 inhibition not only suppresses respiration (Cunningham et al., 2007; Morita et al., 2013) but also elevates mROS during B cell activation. We have revealed mROS accumulation and heme homeostasis as mechanistic connections between mTORC1 signaling and plasma cell differentiation. We speculate that heme accumulation constitutes an intrinsic part of metabolic reprogramming in WT B cells: PpIX accumulation increases concurrently with that of two mTORC1 targets, CD71 and *Hmox1* (Cunningham et al., 2007; Yang et al., 2013). Heme exhibits anti-oxidant properties through hemoxygenase-dependent degradation (Ryter and Tyrrell, 2000), which could also be relevant in this setting.

In terms of the mode of metabolic reprogramming, our results provided further evidence that B cells increase glycolytic flux upon activation (Garcia-Manteiga et al., 2011; Wang et al., 2011) and that PKCβ plays a role in regulating these changes (Blair et al., 2012). Although respiratory rate might not directly affect cell fate in B cells (Jang et al., 2015), metabolic status can heavily influence other downstream pathways through the supply of metabolites derived. In line with this, our metabolomics results indicate that activated *Prkcb*$^{-/-}$ B cells exhibit a shift toward oxidative phosphorylation in compared to WT cells. While this may be beneficial to activated *Prkcb*$^{-/-}$ B cells to supplement ATP production, it may divert metabolites from other biosynthetic processes necessary for cell growth and differentiation, such as succinyl-CoA that is required for heme biosynthesis. Additionally, mitochondrial status may influence the localization of heme biosynthetic enzymes. Thus, our results reflect that changes in mitochondria during B cell activation likely sustain the mTORC1-BACH2-BLIMP1 program through biosynthesis of heme.

How mTORC1 signaling is coupled to PKCβ is not addressed in this study. In mammalian cells, mTORC1 signaling is regulated by both PI3K-dependent and PI3K-independent mechanisms (Laplante and Sabatini, 2013). Notably, mTORC1 activity can be altered by nutrient availability, such as glucose or amino acids (Donahue and Fruman, 2007). Nutrient availability also affects lysosome positioning, which also influence mTORC1 activity (Korolchuk et al., 2011). Reliance on each of these regulatory pathways is context specific (Donahue and Fruman, 2007). Indeed, we observed a strong correlation between the activity of mTORC1 and expression of CD98 in WT B cells, suggesting that the regulation of mTORC1 may switch from PI3K dependent to PI3K independent at some point during B cell activation. While the use of fully supplemented media in our *in vitro* assays unlikely restricts nutrient availability, mTORC1 activity may be affected by altered surface expression of nutrient transporters such as GLUT1 or CD98 in *Prkcb*$^{-/-}$ B cells (Caro-Maldonado et al., 2014; Sinclair et al., 2013). It is also possible that mTORC1 defects in *Prkcb*$^{-/-}$ B cells are a result of lysosome misplacement (Korolchuk et al., 2011; Siemasko et al., 1998). These would be in line with the role of PKCβ in vesicular trafficking described in this study and previously (Kermorgant et al., 2003). Moreover, the lack of *Prdm1* induction in *Prkcb*$^{-/-}$ B cells can further suppress mTORC1 signaling through the loss of positive feedback (Tellier et al., 2016).

Figure 7. Early mTORC1 Signaling Plays a Decisive Role in Effector Fate Decision in B Cells through Heme Metabolism

(A–C) GFP expression, p-S6 (A), cell blasting (B), and CD98 and CD71 surface expression (C) as measured by flow cytometry of *Prdm1*$^{+/GFP}$ B cells cultured in LPS, IL-4, and IL-5 for 2 days in the presence of rapamycin or hemin (added on day 1).

(D) A proposed model relating mTORC1, BACH2, and BLIMP1.

(E) p-S6, CD98, and CD71 expression as determined using flow cytometry of *Prkcb*$^{-/-}$ B cells cultured in LPS, IL-4, and IL-5 for 2 days in the absence or presence of hemin (added on day 1). Data are representative of at least 2 independent experiments.

(F) S6 phosphorylation, CD98, and CD71 surface expression as measured by flow cytometry of WT. Data are representative of 2 independent experiments.

(G) Quantification of CD98 surface expression in *Cd19*$^{-/-}$ B cells cultured in LPS, IL-4, and IL-5 for 2 days in the absence or presence of hemin (added on day 1). Data are representative of 2 independent experiments.

(H) Expressions of PAX5, IRF4, CD138, and IgG1 and the corresponding quantifications as analyzed by flow cytometry of WT and *Cd19*$^{-/-}$ B cells cultured in LPS, IL-4, and IL-5 for 3 days in the absence or presence of hemin (added on day 1). Data were analyzed using two-way ANOVA and are representative of 2 independent experiments.

Error bars represent SEM.

An increasing body of evidence suggests that mTORC1 signaling and metabolic reprogramming are key determinants for lymphocyte fate decisions. In activated T cells, mTORC1 signaling cooperates with Myc to determine effector functions (Pollizzi et al., 2016; Verbist et al., 2016). We speculate that much of these also apply to B cells. Indeed, systems previously reported to exhibit defective PI3K or mTORC1 signaling, such as *Cdc42*$^{-/-}$ and *Wipf*$^{-/-}$, all display altered "effector" versus "memory" fate decisions (Burbage et al., 2015; Keppler et al., 2015). With the inclusion of the *Prkcb*$^{-/-}$ and *Cd19*$^{-/-}$ model presented in this study, there is a convincing body of evidence that illustrates the decisiveness of mTORC1 signaling axis in steering fate decision in B cells (Benhamron et al., 2015; Kometani et al., 2013). Given that the dependence on mTORC1 seems to be transient, we speculate that early mTORC1 activity may initiate a crosstalk with BACH2, BLIMP1, and metabolic reprogramming, which later becomes a self-propagating program via positive feedback mechanisms to promote plasma cell differentiation. This would be in line with the behavior and function of mTORC1 activity in GC B cells (Ersching et al., 2017). Finally, we have identified the crucial role of PKCβ in promoting early mTORC1 signaling and regulating transcriptomic and metabolic reprogramming, which together instruct and maintain effector function in activated B cells.

STAR★METHODS

Detailed methods are provided in the online version of this paper and include the following:

- KEY RESOURCES TABLE
- CONTACT FOR REAGENT AND RESOURCE SHARING
- EXPERIMENTAL MODELS
  - Animal breeding and generation
  - Immunization, infection, ELISA and ELISPOT
  - Cell isolation, labeling and culture
- METHOD DETAILS
  - Proliferation analysis
  - Antigen internalization and presentation
  - Microspheres preparation
  - Eα Peptide
  - Flow cytometry
  - PpIX measurement
  - Immunoblotting
  - Optical microscopy
  - RNA sequencing and bioinformatics analysis
  - Metabolomics fingerprinting
  - Extracellular flux assay
  - U-^{13}C labeling assay
- QUANTIFICATION AND STATISTICAL ANALYSIS

SUPPLEMENTAL INFORMATION

Supplemental Information contains six figures and one table and can be found with this article online at https://doi.org/10.1016/j.immuni.2018.04.031.

ACKNOWLEDGMENTS

We thank Daisuke Kitamura (Tokyo University of Sciences) for 40LB cells. We thank Dinis Calado (The Francis Crick Institute) for advice and suggestions. We thank the BRF and flow cytometry unit (The Francis Crick Institute) for animal maintenance and technical support. We thank the Advanced Sequencing and Bioinformatics (The Francis Crick Institute) for assistance. We thank all members of the Lymphocyte Interaction Laboratory (The Francis Crick Institute and Ragon Institute) for critical comments. All work presented here was supported by the Francis Crick Institute core funded by CRUK (FC001035and FC001136), the UK Medical Research Council (FC001035 and FC001136), Wellcome Trust (FC001035 and FC001136), UCL ORS Award to C.T., a Marie Sklodowska-Curie individual postdoctoral fellowship to N.M.-M., the center for HIV/AIDS Vaccine Immunology and Immunogen Discovery of the National Institutes of Health (NIH; UM1AI100663), the Philip T. and Susan M. Ragon Institute Foundation, and the Bill and Melinada Gates Foundation Innovation Award (228966) to F.D.B.

AUTHOR CONTRIBUTIONS

C.T. and N.M.-M. designed and carried out the experiments. M.G. and P.M. performed VACV and immunization experiments. M. Llorian analyzed RNA-seq data. N.M.L., M.R., and J.I.M. performed metabolomics assays. A.J.C. and P.J.P. advised on the project. M. Leitges provided the *Prkca*$^{-/-}$ and *Prkcb*$^{-/-}$ lines. A.B. performed image analyses. F.D.B. supervised the project. C.T. and F.D.B. wrote the paper.

DECLARATION OF INTERESTS

The authors declare no competing interests.

Received: October 12, 2017
Revised: March 13, 2018
Accepted: April 27, 2018
Published: June 5, 2018

REFERENCES

Ajioka, R.S., Phillips, J.D., and Kushner, J.P. (2006). Biosynthesis of heme in mammals. Biochim. Biophys. Acta *1763*, 723–736.

Batista, F.D., and Harwood, N.E. (2009). The who, how and where of antigen presentation to B cells. Nat. Rev. Immunol. *9*, 15–27.

Benhamron, S., Pattanayak, S.P., Berger, M., and Tirosh, B. (2015). mTOR activation promotes plasma cell differentiation and bypasses XBP-1 for immunoglobulin secretion. Mol. Cell. Biol. *35*, 153–166.

Blair, D., Dufort, F.J., and Chiles, T.C. (2012). Protein kinase Cβ is critical for the metabolic switch to glycolysis following B-cell antigen receptor engagement. Biochem. J. *448*, 165–169.

Buhl, A.M., Pleiman, C.M., Rickert, R.C., and Cambier, J.C. (1997). Qualitative regulation of B cell antigen receptor signaling by CD19: selective requirement for PI3-kinase activation, inositol-1,4,5-trisphosphate production and Ca2+ mobilization. J. Exp. Med. *186*, 1897–1910.

Burbage, M., Keppler, S.J., Gasparrini, F., Martínez-Martín, N., Gaya, M., Feest, C., Domart, M.-C., Brakebusch, C., Collinson, L., Bruckbauer, A., and Batista, F.D. (2015). Cdc42 is a key regulator of B cell differentiation and is required for antiviral humoral immunity. J. Exp. Med. *212*, 53–72.

Caro-Maldonado, A., Wang, R., Nichols, A.G., Kuraoka, M., Milasta, S., Sun, L.D., Gavin, A.L., Abel, E.D., Kelsoe, G., Green, D.R., and Rathmell, J.C. (2014). Metabolic reprogramming is required for antibody production that is suppressed in anergic but exaggerated in chronically BAFF-exposed B cells. J. Immunol. *192*, 3626–3636.

Chaturvedi, A., Dorward, D., and Pierce, S.K. (2008). The B cell receptor governs the subcellular location of Toll-like receptor 9 leading to hyperresponses to DNA-containing antigens. Immunity *28*, 799–809.

Cunningham, J.T., Rodgers, J.T., Arlow, D.H., Vazquez, F., Mootha, V.K., and Puigserver, P. (2007). mTOR controls mitochondrial oxidative function through a YY1-PGC-1α transcriptional complex. Nature *450*, 736–740.

De Silva, N.S., and Klein, U. (2015). Dynamics of B cells in germinal centres. Nat. Rev. Immunol. *15*, 137–148.

Donahue, A.C., and Fruman, D.A. (2007). Distinct signaling mechanisms activate the target of rapamycin in response to different B-cell stimuli. Eur. J. Immunol. *37*, 2923–2936.

Eckl-Dorna, J., and Batista, F.D. (2009). BCR-mediated uptake of antigen linked to TLR9 ligand stimulates B-cell proliferation and antigen-specific plasma cell formation. Blood *113*, 3969–3977.

Ersching, J., Efeyan, A., Mesin, L., Jacobsen, J.T., Pasqual, G., Grabiner, B.C., Dominguez-Sola, D., Sabatini, D.M., and Victora, G.D. (2017). Germinal center selection and affinity maturation require dynamic regulation of mTORC1 kinase. Immunity *46*, 1045–1058.e6.

Garcia-Manteiga, J.M., Mari, S., Godejohann, M., Spraul, M., Napoli, C., Cenci, S., Musco, G., and Sitia, R. (2011). Metabolomics of B to plasma cell differentiation. J. Proteome Res. *10*, 4165–4176.

Jang, K.-J., Mano, H., Aoki, K., Hayashi, T., Muto, A., Nambu, Y., Takahashi, K., Itoh, K., Taketani, S., Nutt, S.L., et al. (2015). Mitochondrial function provides instructive signals for activation-induced B-cell fates. Nat. Commun. *6*, 6750.

Kallies, A., Hasbold, J., Tarlinton, D.M., Dietrich, W., Corcoran, L.M., Hodgkin, P.D., and Nutt, S.L. (2004). Plasma cell ontogeny defined by quantitative changes in blimp-1 expression. J. Exp. Med. *200*, 967–977.

Keppler, S.J., Gasparrini, F., Burbage, M., Aggarwal, S., Frederico, B., Geha, R.S., Way, M., Bruckbauer, A., and Batista, F.D. (2015). Wiskott-Aldrich syndrome interacting protein deficiency uncovers the role of the co-receptor CD19 as a generic hub for PI3 kinase signaling in B cells. Immunity *43*, 660–673.

Kermorgant, S., Zicha, D., and Parker, P.J. (2003). Protein kinase C controls microtubule-based traffic but not proteasomal degradation of c-Met. J. Biol. Chem. *278*, 28921–28929.

Klein, U., Casola, S., Cattoretti, G., Shen, Q., Lia, M., Mo, T., Ludwig, T., Rajewsky, K., and Dalla-Favera, R. (2006). Transcription factor IRF4 controls plasma cell differentiation and class-switch recombination. Nat. Immunol. *7*, 773–782.

Kometani, K., Nakagawa, R., Shinnakasu, R., Kaji, T., Rybouchkin, A., Moriyama, S., Furukawa, K., Koseki, H., Takemori, T., and Kurosaki, T. (2013). Repression of the transcription factor Bach2 contributes to predisposition of IgG1 memory B cells toward plasma cell differentiation. Immunity *39*, 136–147.

Korolchuk, V.I., Saiki, S., Lichtenberg, M., Siddiqi, F.H., Roberts, E.A., Imarisio, S., Jahreiss, L., Sarkar, S., Futter, M., Menzies, F.M., et al. (2011). Lysosomal positioning coordinates cellular nutrient responses. Nat. Cell Biol. *13*, 453–460.

Laplante, M., and Sabatini, D.M. (2013). Regulation of mTORC1 and its impact on gene expression at a glance. J. Cell Sci. *126*, 1713–1719.

Leitges, M., Schmedt, C., Guinamard, R., Davoust, J., Schaal, S., Stabel, S., and Tarakhovsky, A. (1996). Immunodeficiency in protein kinase cbeta-deficient mice. Science *273*, 788–791.

Li, M., Davey, G.M., Sutherland, R.M., Kurts, C., Lew, A.M., Hirst, C., Carbone, F.R., and Heath, W.R. (2001). Cell-associated ovalbumin is cross-presented much more efficiently than soluble ovalbumin in vivo. J. Immunol. *166*, 6099–6103.

MacRae, J.I., Dixon, M.W., Dearnley, M.K., Chua, H.H., Chambers, J.M., Kenny, S., Bottova, I., Tilley, L., and McConville, M.J. (2013). Mitochondrial metabolism of sexual and asexual blood stages of the malaria parasite Plasmodium falciparum. BMC Biol. *11*, 67.

Martínez-Martín, N., Maldonado, P., Gasparrini, F., Frederico, B., Aggarwal, S., Gaya, M., Tsui, C., Burbage, M., Keppler, S.J., Montaner, B., et al. (2017). A switch from canonical to noncanonical autophagy shapes B cell responses. Science *355*, 641–647.

McHeyzer-Williams, L.J., and McHeyzer-Williams, M.G. (2005). Antigen-specific memory B cell development. Annu. Rev. Immunol. *23*, 487–513.

Mellor, H., and Parker, P.J. (1998). The extended protein kinase C superfamily. Biochem. J. *332*, 281–292.

Morita, M., Gravel, S.-P., Chénard, V., Sikström, K., Zheng, L., Alain, T., Gandin, V., Avizonis, D., Arguello, M., Zakaria, C., et al. (2013). mTORC1 controls mitochondrial activity and biogenesis through 4E-BP-dependent translational regulation. Cell Metab. *18*, 698–711.

Muramatsu, M., Kinoshita, K., Fagarasan, S., Yamada, S., Shinkai, Y., and Honjo, T. (2000). Class switch recombination and hypermutation require activation-induced cytidine deaminase (AID), a potential RNA editing enzyme. Cell *102*, 553–563.

Muto, A., Tashiro, S., Nakajima, O., Hoshino, H., Takahashi, S., Sakoda, E., Ikebe, D., Yamamoto, M., and Igarashi, K. (2004). The transcriptional programme of antibody class switching involves the repressor Bach2. Nature *429*, 566–571.

Nojima, T., Haniuda, K., Moutai, T., Matsudaira, M., Mizokawa, S., Shiratori, I., Azuma, T., and Kitamura, D. (2011). In-vitro derived germinal centre B cells differentially generate memory B or plasma cells in vivo. Nat. Commun. *2*, 465.

Nutt, S.L., Hodgkin, P.D., Tarlinton, D.M., and Corcoran, L.M. (2015). The generation of antibody-secreting plasma cells. Nat. Rev. Immunol. *15*, 160–171.

Pollizzi, K.N., Sun, I.-H., Patel, C.H., Lo, Y.-C., Oh, M.-H., Waickman, A.T., Tam, A.J., Blosser, R.L., Wen, J., Delgoffe, G.M., and Powell, J.D. (2016). Asymmetric inheritance of mTORC1 kinase activity during division dictates CD8(+) T cell differentiation. Nat. Immunol. *17*, 704–711.

Rajewsky, K. (1996). Clonal selection and learning in the antibody system. Nature *381*, 751–758.

Rudensky, A.Y., Preston-Hurlburt, P., Hong, S.C., Barlow, A., and Janeway, C.A., Jr. (1991). Sequence analysis of peptides bound to MHC class II molecules. Nature *353*, 622–627.

Ryter, S.W., and Tyrrell, R.M. (2000). The heme synthesis and degradation pathways: role in oxidant sensitivity. Heme oxygenase has both pro- and antioxidant properties. Free Radic. Biol. Med. *28*, 289–309.

Saijo, K., Mecklenbräuker, I., Santana, A., Leitger, M., Schmedt, C., and Tarakhovsky, A. (2002). Protein kinase C beta controls nuclear factor kappaB activation in B cells through selective regulation of the IkappaB kinase alpha. J. Exp. Med. *195*, 1647–1652.

Sciammas, R., Shaffer, A.L., Schatz, J.H., Zhao, H., Staudt, L.M., and Singh, H. (2006). Graded expression of interferon regulatory factor-4 coordinates isotype switching with plasma cell differentiation. Immunity *25*, 225–236.

Shapiro-Shelef, M., Lin, K.-I., McHeyzer-Williams, L.J., Liao, J., McHeyzer-Williams, M.G., and Calame, K. (2003). Blimp-1 is required for the formation of immunoglobulin secreting plasma cells and pre-plasma memory B cells. Immunity *19*, 607–620.

Shinnakasu, R., Inoue, T., Kometani, K., Moriyama, S., Adachi, Y., Nakayama, M., Takahashi, Y., Fukuyama, H., Okada, T., and Kurosaki, T. (2016). Regulated selection of germinal-center cells into the memory B cell compartment. Nat. Immunol. *17*, 861–869.

Siemasko, K., Eisfelder, B.J., Williamson, E., Kabak, S., and Clark, M.R. (1998). Cutting edge: signals from the B lymphocyte antigen receptor regulate MHC class II containing late endosomes. J. Immunol. *160*, 5203–5208.

Sinclair, L.V., Rolf, J., Emslie, E., Shi, Y.-B., Taylor, P.M., and Cantrell, D.A. (2013). Control of amino-acid transport by antigen receptors coordinates the metabolic reprogramming essential for T cell differentiation. Nat. Immunol. *14*, 500–508.

Su, T.T., Guo, B., Kawakami, Y., Sommer, K., Chae, K., Humphries, L.A., Kato, R.M., Kang, S., Patrone, L., Wall, R., et al. (2002). PKC-β controls I κ B kinase lipid raft recruitment and activation in response to BCR signaling. Nat. Immunol. *3*, 780–786.

Tellier, J., Shi, W., Minnich, M., Liao, Y., Crawford, S., Smyth, G.K., Kallies, A., Busslinger, M., and Nutt, S.L. (2016). Blimp-1 controls plasma cell function through the regulation of immunoglobulin secretion and the unfolded protein response. Nat. Immunol. *17*, 323–330.

Verbist, K.C., Guy, C.S., Milasta, S., Liedmann, S., Kamiński, M.M., Wang, R., and Green, D.R. (2016). Metabolic maintenance of cell asymmetry following division in activated T lymphocytes. Nature *532*, 389–393.

Victora, G.D., and Nussenzweig, M.C. (2012). Germinal centers. Annu. Rev. Immunol. *30*, 429–457.

Vinuesa, C.G., and Cyster, J.G. (2011). How T cells earn the follicular rite of passage. Immunity *35*, 671–680.

Wang, R., Dillon, C.P., Shi, L.Z., Milasta, S., Carter, R., Finkelstein, D., McCormick, L.L., Fitzgerald, P., Chi, H., Munger, J., and Green, D.R. (2011). The transcription factor Myc controls metabolic reprogramming upon T lymphocyte activation. Immunity *35*, 871–882.

Watanabe-Matsui, M., Muto, A., Matsui, T., Itoh-Nakadai, A., Nakajima, O., Murayama, K., Yamamoto, M., Ikeda-Saito, M., and Igarashi, K. (2011). Heme regulates B-cell differentiation, antibody class switch, and heme oxygenase-1 expression in B cells as a ligand of Bach2. Blood *117*, 5438–5448.

Yang, K., Shrestha, S., Zeng, H., Karmaus, P.W.F., Neale, G., Vogel, P., Guertin, D.A., Lamb, R.F., and Chi, H. (2013). T cell exit from quiescence and differentiation into Th2 cells depend on Raptor-mTORC1-mediated metabolic reprogramming. Immunity *39*, 1043–1056.

STAR★METHODS

KEY RESOURCES TABLE

REAGENT or RESOURCE	SOURCE	IDENTIFIER
Antibodies		
Anti-B220	eBioscience	Clone: [RA3-6B2]
Anti-CD138	BIoLegend	Clone: [281.2]
Anti-CD16/32	eBioscience	CAT: 14-0161-86
Anti-CD19	eBioscience	Clone: [eBio-1D3]
Anti-CD4	BIoLegend	Clone: [GK1.5]
Anti-CD44	eBioscience	Clone: [IM7]
Anti-CD71	BD Biosciences	Clone: [C2]
Anti-CD95	BD Biosciences	Clone: [Jo2]
Anti-CD98	BioLegend	Clone: [RL388]
Anti-CXCR5	BD Biosciences	Clone: [2G8]
Anti-Erk	Cell Signaling Tech	clone: [137F5]
Anti-GL7	BD Biosciences	Clone: [GL7]
Anti-GLUT1	Abcam	Clone: [SPM498]
Anti-IgD	BD Biosciences	Clone: [11-26c.2a]
Anti-IgG biotinylated	Southern Biotech	CAT: 1030-08
Anti-IgG1	BD Biosciences	Clone: [A85.1]
Anti-IgM biotinylated	Southern Biotech	CAT: 1020-08
Anti-IRF4	BioLegend	Clone: [IRF4.3E]
Anti-kappa	BD Biosciences	Clone: [187.1]
Anti-MHCII:Ea	eBioscience	Clone: [eBioY-Ae]
Anti-Mouse-IgG HRP	Jackson ImmunoResearch	CAT: 115-035-003
Anti-Mouse-IgG2b	Life Technologies	Clone: [RMG2b-1]
Anti-p-Akt	Cell Signaling Tech	clone: [D9E]
Anti-p-S6	Cell Signaling Tech	Clone: [D57.2.2E]
Anti-p-S6K1	Cell Signaling Tech	Clone: [108D2]
Anti-PAX5	BioLegend	Clone: [IH9]
Anti-PD1	eBioscience	Clone: [J43]
Anti-Rabbit-IgG	Life Technologies	CAT: A-11008
Anti-Rabbit-IgG HRP	Jackson ImmunoResearch	CAT: 111-035-144
Chemicals, Peptides, and Recombinant Proteins		
CD40L	R&D Systems	CAT: 1163-CL
CFSE	Invitrogen	CAT: C34570
Chloroform Optima grade (for HPLC, stabilized with Amylene)	FISHER Chemicals UK	N/A
CpG (ODN 1826)	Sigma	N/A
CellTrace violet (CTV)	Invitrogen	CAT: C34557
Eα peptide	Internal source	N/A
FCCP	Sigma	CAT: C2920
Gö 6976	Calbiochem	CAT: 365250
Hemin	Sigma	CAT: 9039
Interleukin-4	R&D Systems	404-ML
Interleukin-5	R&D Systems	405-ML
L-ascorbic acid	Sigma	CAT: A92902
LPS	Sigma	CAT: L3012
MeOH Optima grade	FISHER Chemicals UK	N/A

(*Continued on next page*)

Continued

REAGENT or RESOURCE	SOURCE	IDENTIFIER
Mitoqunione	FOCUS Biomolecules	CAT: 10-1363
MitoSOX	Life Technologies	CAT: M36008
MitoTempo	Sigma	CAT: SML0737
MitoTracker Green	Life Technologies	CAT: M7514
MitotTracker Red CMXRos	Cell Signaling Tech	CAT: 9082S
Mouse B cell isolation kit	Miltenyi	CAT: 130-090-862
Mouse CD4 T cell isolation kit	Miltenyi	CAT: 130-104-454
Nocodazole	Calbiochem	CAT: 487928
Oligomycin A	Sigma	CAT: 75351
Rotenone	Sigma	CAT: R8875
Water Optima grade	FISHER Chemicals UK	N/A
Deposited Data		
RNA sequencing data	This paper	GEO: GSE111702
Experimental Models: Organisms/Strains		
Prkca$^{-/-}$	Michael Leitges (University of Oslo)	N/A
Prkcb$^{-/-}$	Michael Leitges (University of Oslo)	N/A
Prdm1$^{+/GFP}$	Internal source	N/A
Cd19$^{-/-}$	Internal source	N/A

CONTACT FOR REAGENT AND RESOURCE SHARING

Further information and requests for resources and reagents should be directed to and will be fulfilled by the Lead Contact, Facundo D. Batista (Fbatista1@mgh.harvard.edu).

EXPERIMENTAL MODELS

Animal breeding and generation

PKCβ-deficient and PKCα-deficient mice were kindly provided by Michael Leigtes, Oslo. All mice were bred and maintained at the animal facility of Cancer Research UK and The Francis Crick Institute. The Animal Ethics Committee of Cancer Research UK, The Francis Crick Institute and the UK Home Office approved all experiments.

Immunization, infection, ELISA and ELISPOT

For immunization, mice were injected intra-peritoneally with 50 μg NP_{23}-KLH (Biosearch Technology) in 4 mg Alum (ThermoScientific). Blood samples were taken from the lateral tail-vein on day 0, 3, 7, 13, 28 after immunization. For infection, 10^4 PFU of Vaccinia Virus Western Reserve strain (vacv) was injected into isoflurane-anesthetized animals in the footpads. NP-specific antibody titers were detected by ELISA, using NP_{23}-BSA, NP_3-BSA, and biotinylated anti–mouse IgM and IgG (Southern Biotech). Titers were determined from the dilution curve in the linear range of absorbance. All non-commercial ELISA plates were developed with alkaline-phosphatase streptavidin (Sigma) and phosphorylated nitrophenyl-phosphate (Sigma). Absorbance at 405 nm was determined with a SPECTRAmax190 plate reader (Molecular Devices). NP-specific antibody-secreting cells and vacv-specific antibody-secreting cells were captured using activated ELISPOT plates coated with either NP_{23}-BSA or vacv respectively. Detection was made using biotinylated anti–mouse IgM (Southern Biotech), IgG (Southern Biotech). All ELISPOT plates were developed with alkaline-phosphatase streptavidin (Sigma) and the BCiP®/NBT reaction (Sigma). Images were acquired using CTL 4.0 (ImmunoSpot®).

Cell isolation, labeling and culture

Splenic naive B or CD4 T lymphocytes were purified using negative B cell or CD4 T cell isolation kits yielding enriched populations of ~95%–98% (B cells) and ~80% (T cells), respectively (Miltenyi Biotec). Purified B or T cells were labeled in PBS with 2 μM CTV (Invitrogen) or 1 μM CFSE (Invitrogen) for 5 minutes at 37°C with 5% CO_2. Cells were maintained in complete B cell medium (RPMI 1640 supplemented with 10% FCS, 25 mM HEPES, Glutamax, penicillin and streptomycin (Invitrogen), and 1% β-mercaptoethanol (Sigma)). For iGC feeder (40LB) cells, cells were cultured in DMEM supplemented with 10% FCS and penicillin and streptomycin (Invitrogen). Initial selection for CD40L- and BAFF-expressing cells was performed using G418 and puromycin as described (Nojima et al., 2011).

METHOD DETAILS

Proliferation analysis

CFSE- or CTV–labeled cells at a concentration of 10^6 cells per mL were stimulated in complete B cell medium supplemented with combinations of 1 μg/mL LPS (Sigma) or 0.05 μg/ml CD40L (R&D Systems), 5 μg/ml anti-IgM F(ab)$_2$ fragment (Jackson ImmunoResearch), 1.5 ug/mL CpG (Sigma) 10 ng/ml of IL-4 (R&D Systems), or 10 ng/ml of IL-5 (R&D Systems). CFSE or CTV dilution was measured after 3 or 4 days by flow cytometry. Various agents were added as follow: L-ascorbic acid (200 μM) on day1, Mitoqunione of specified concentrations, MitoTempo of specified concentrations, hemin (60 μM) on day 1 and Rapamycin (50 nM) on the specified time points.

Antigen internalization and presentation

For internalization assays, purified B cells were loaded with IgM-coated beads on ice for 60 minutes. Cells were then washed with complete medium to remove excess antigen, and then incubated for 30 minutes at 37°C. Cells were fixed at different time points with 4% formaldehyde. After fixation, beads remaining on the cell surface were detected with Alexa488 streptavidin (eBioscience). To detect antigen presentation, B cells loaded with Eα peptide and IgM-coated beads were incubated between 3 and 5 hours at 37°C, and then fixed in 4% formaldehyde. These cells were then stained with anti-MHCII:Eα antibody, followed by anti–mouse IgG2b antibody staining for detection by flow cytometry.

Microspheres preparation

For proliferation assay, 0.11 μm (diameter) streptavidin-coated microspheres (Bangs Laboratories) were incubated with a saturating amount of biotinylated anti-IgM and biotinylated-CpG (Sigma) or OVA (EMD Millipore) for 1 hour at 37°C, and washed to removed unbound molecules. For presentation assays, 0.11 μm red microspheres (Bangs Laboratories) were incubated with a saturating amount of biotinylated anti-IgM (Southern Biotech) and Eα peptide or OVA (EMD Millipore) for 1 hour at 37°C. Limiting stimulatory conditions were obtained by increasing the amounts of OVA or Eα peptide for coating, whereas IgM amounts were kept constant. Efficient titration of the IgM signal was measured by flow cytometry. Beads coated with anti-IgM were used as negative control. Red microspheres (Bangs Laboratories) coated with biotinylated anti-IgM (Southern Biotech) were used for internalization assay.

Eα Peptide

Biotin-GSGFAKFASFEAQGALANIAVDKA-COOH was produced by the Crick Peptide Chemistry facility.

Flow cytometry

For analysis of splenocyte populations, single-cell suspensions were prepared from homogenized spleens. Erythrocytes were destroyed with Lysis Buffer (BD Biosciences). Cells were treated with the appropriate combination of the following antibodies: CD16/32 (Fc block), B220, CD19, CD44, PD1, IgG1, IgD, CD95, GL7, CXCR5, CD4, and CD138. For analysis of *in vitro* B cell cultures, after blocking Fc receptors using anti-CD16/32 antibodies, CTV-labeled cells were stained with the antibodies CD138, IgG1, CD98 and CD71. For intracellular detection of PAX5, IRF4, p-S6K1, p-S6 and GLUT1, after blocking Fc receptors using anti-CD16/32 antibodies, cells were fixed and permeablized with Cytofix/Cytoperm (BD Biosciences). Antibody against PAX5 and IRF4 diluted in 1x Perm/Wash (BD Biosciences) were used. Primary antibody against p-S6K1, p-S6, GLUT1 and secondary Alexa488 or Alexa555-conjugated Goat-anti-Rabbit IgG antibody (Life Technologies) was used for their detection. Mitochondrial status was measured using MitoTracker Green (20 nM), MitoTracker Red CMXRos (20 nM) and MitoSOX (5 μM). Cells were labeled for 30 minutes at 37°C. Cells were washed once with 2% FCS supplemented PBS and analyzed by flow cytometry. The relative mitochondrial quality was calculated by normalizing the intensity (MFI) of MitoTracker Red CMXRos to the intensity (MFI) of MitoTracker Green. Data were acquired on LSR Fortessa (BD) and analyzed with FlowJo (Tree Star).

PpIX measurement

Cells were analyzed using flow cytometry. Excitation at 405nm and emission at 605/40 nm were used.

Immunoblotting

Purified B cells were left at 37°C for 10 minutes in Imaging buffer (PBS, 0.5% FCS, 1 g/L D-Glucose, 2 mM MgCl2, and 0.5 mM $CaCl_2$) to equilibrate before stimulation. They were then stimulated for various times with 5 μg/ml anti-IgM F(ab)$_2$ fragment (Jackson ImmunoResearch) and 1.5 ug/mL CpG, 10 ng/ml of IL4, 10 ng/ml of IL-5, or coated microspheres (see previous section). For immunoblotting, stimulated cells were then lysed in lysis buffer (20 mM Tris-HCL, pH 8.0, 150 mM NaCl, 5 mM EDTA, Protease Inhibitor cocktail (Roche), 10 mM NaF, 1 mM Na_3VO_4, and 1% NP-40) for 30 minutes on ice, and samples were loaded into 12% PAGE gel (BIO-RAD) for electrophoresis. Proteins were detected with antibodies against p-Akt (Ser473), p-S6k1 (Thr389) and Erk using the secondary HRP-conjugated anti–rabbit or anti–mouse antibodies (see Key Resources Table). Blot densitometry analysis was performed using the ImageJ (National Institutes of Health) software.

Optical microscopy

Spleens were embedded in OCT and frozen in cold isopentane and 10 μm-wide frozen sections were cut with a cryostat. Sections were dehydrated and fixed in 4% paraformaldehyde, blocked with PBS containing 1% BSA, and 10% goat serum (IF blocking buffer). To label plasma cell population architecture, sections were also permeablized with PBS 0.3% Triton for 3 minutes. Staining was performed in IF blocking buffer with a combination of the following antibodies: B220, anti-κ, and GL7. Confocal imaging was performed with a LSM 780 microscope (Carl Zeiss) with a plan apochromat 20 ×, NA 0.8 objective for tissue sections or a plan apochromat 63 ×, NA 1.40 objective for other applications. Images were analyzed with Imaris (Bitplane) or ImageJ software. For tissue sections, tiled images were acquired and assembled with the Zen software.

RNA sequencing and bioinformatics analysis

RNA from B cells were extracted and purified with MagMAX^{TM} RNA isolation kit (Life Technologies). Samples were processed with KAPA hyper prep and sequenced using the HiSeq 4000 system. Sequencing on biological triplicates (WT) and duplicates (*Prkcb*$^{-/-}$) generated libraries ranging 40-70 million, 101 bp paired end reads. Read trimming and adaptor-removal were performed using Trim Galore! (version 0.4.2). The RSEM package (version 1.2.31), and Bowtie2 (version 2.2.9) were used to align reads to the mouse genome (Ensembl GRCm38 release 85) and to obtain gene level counts. For RSEM, all parameters were run as default except '–forward-prob' that was set to '0'. Differential expression analysis was carried out with DESeq2 package (version 1.14.1) within R version 3.3.2. Genes were considered to be differential expressed with $p_{adj} \leq 0.05$. Gene set enrichment Analysis (GSEA) (version 2.2.3) was done for each pairwise comparison using gene lists ranked using the Wald statistic. Gene set pre-ranked analysis was carried out with respect of gene sets C2 canonical pathways and C5 biological processes. All parameters were kept as default except for enrichment statistic (classic) and min/max size, which were changed to 5 and 50000 respectively. Gene signatures with FDR q-value ≤ 0.05 were considered significant. Heatmap of differential expressed genes belonging to gene ontologies; mitochondrion (GO:0005739) were generated using the gplots CRAN package (version 3.0.1). Genes were clustered using an Eisen distance matrix and average linkage clustering.

Metabolomics fingerprinting

Polar metabolites were extracted and analyzed by GC-MS as follows: 5 μL culture supernatant was removed from each sample and polar metabolites were phase-partitioned from apolar metabolites by addition of 350 μL chloroform/methanol/water (1:3:3 v/v/v, containing 1 nmol *scyllo*-inositol as internal standard). Centrifugation (13,000 rpm, 10 mins, 4°C) was used to separate phases. The polar phase dried in a rotary vacuum concentrator and washed twice with methanol. Metabolites were analyzed as previously described (MacRae et al., 2013). In brief, metabolites were derivatized by methoximation [20 μl 20 mg/ml methoxyamine-HCl (Sigma, 226904) in pyridine (Sigma, 270970) at RT, overnight], and subsequent incubation with 20 μl N,O-bis(trimetylsilyl)trifluoroacetamide (BSTFA) + 1% trimethylchlorosilane (TMCS) (Sigma, 33148) for ≥ 1 hr. Metabolite analysis was performed by GC-MS using an Agilent 7890B-5977A system. Splitless injection (injection temperature 270°C) onto a 30 m + 10 m × 0.25 mm DB-5MS+DG column (Agilent J&W) was used, with helium as the carrier gas, in electron impact ionization (EI) mode. The initial oven temperature was 70°C (2 min), followed by temperature gradients to 295°C at 12.5°C/min and then to 320°C 25°C/min (held for 3 mins). Metabolites were identified and quantified by comparison to the retention times, mass spectra, and responses of known amounts of authentic standards using MassHunter Workstation software (B.06.00 SP01, Agilent Technologies).

Extracellular flux assay

Naive and activated B cells were resuspended in Seahorse medium supplemented with 11 mM glucose and 2 mM pyruvate with pH adjusted to 7.4. Cells were settled on 96-well assay plate (Seahorse Bioscience) coated with poly-lysine (Sigma). OCR was recorded with the XF96 Extracellular Flux analyzer. Oligomycin-sensitive OCR represents the difference in OCR before and after addition of 3 μM of Oligomycin A (Sigma). Other chemicals used: FCCP (5 μM) (Sigma); Rotenone (5 μM) (Sigma). Resting ECAR from the same assay was plotted.

U-^{13}C labeling assay

B cells cultured for 2 days were labeled with U-^{13}C-glucose for 2 hours at 37°C. Metabolic activity was quenched rapidly with ethanol/dry ice slurry. Cells were lysed and metabolites were extracted as follows: 600 μL chloroform:methanol (3:1v/v) was added to each sample and vortexed briefly before pulse sonication (3 × 8 minutes) in a water-bath sonicator at 4°C for 1 hour. Samples were spun (13,200 rpm, 4°C, 10 minutes), supernatant transferred to a new tube and dried in a rotary vacuum concentrator. The remaining pellet was re-extracted with 600 μL methanol:water (3:1v/v) followed by pulse sonication for 8 minutes at 4°C. Samples were spun (as above), and the supernatant added to the first extract and dried. Extracts were suspended in 50 μL chloroform and 300 μL methanol:water (1:1) to partition polar and apolar metabolites. Centrifugation (13,000 rpm, 10 minutes, 4°C) was used to separate phases. 150 μL of the polar phase were inserted in a LC-MS vial insert and 50 μL of a mixture of methanol/water (1:1 v/v, containing 1.5 nmol ^{13}C, ^{15}N-Valine as internal standard) were added. Metabolite analysis was performed by LC-MS using a Q-Exactive Plus (Orbitrap) mass spectrometer from Thermo Fisher Scientific (Bremen, Germany) coupled with a Vanquish UHPLC system from Thermo Fisher Scientific (Bremen, Germany). The chromatographic separation was performed on a SeQuant® Zic®-pHILIC (Merck Millipore)

column (5 μm particle size, polymeric, 150 × 4.6 mm). The injection volume was 10 μL, the oven temperature was maintained at 25°C, and the autosampler tray temperature was maintained at 4°C. Chromatographic separation was achieved using a gradient program at a constant flow rate of 300 μl/min over a total run time of 25 min. The elution gradient was programmed as decreasing percentage of B from 80% to 5% during 17 minutes, holding at 5% of B during 3 minutes and finally re-equilibrating the column at 80% of B during 4 minutes. Solvent A was 20 mM ammonium carbonate and 1.4 mL/L of a solution of ammonium hydroxide at 35% in water (pH 9) and solvent B was acetonitrile. Metabolites were identified and quantified by accurate mass and retention time and by comparison to the retention times, mass spectra, and responses of known amounts of authentic standards using TraceFinder 4.1 EFS software (Thermo Fisher Sientific). Label incorporation and abundance was estimated using TraceFinder 4.1 EFS software. The degree of labeling of individual metabolites was estimated as the percentage of the metabolite pool containing one or more ^{13}C atoms after correction for natural abundance isotopes. Abundance was given relatively to the internal standard.

QUANTIFICATION AND STATISTICAL ANALYSIS

Sample sizes were chosen on the basis of published work in which similar phenotypical characterization and similar defects were reported. Cohort randomization or ‘blinding’ of investigators to sample identity was not done in this study. For all statistical comparisons unless specified, the data for each group were compared with Student’s t test and P values were calculated. Normal distribution of samples was assumed on the basis of published studies with analyses similar to ours. Statistically significant differences are indicated on the figures as follows: *$p < 0.05$, **$p < 0.005$, ***$p < 0.0005$, ****$p < 0.00005$.

Immunity, Volume *48*

Supplemental Information

Protein Kinase C-β Dictates B Cell Fate by Regulating Mitochondrial Remodeling, Metabolic Reprogramming, and Heme Biosynthesis

Carlson Tsui, Nuria Martinez-Martin, Mauro Gaya, Paula Maldonado, Miriam Llorian, Nathalie M. Legrave, Merja Rossi, James I. MacRae, Angus J. Cameron, Peter J. Parker, Michael Leitges, Andreas Bruckbauer, and Facundo D. Batista

Supplemental Information

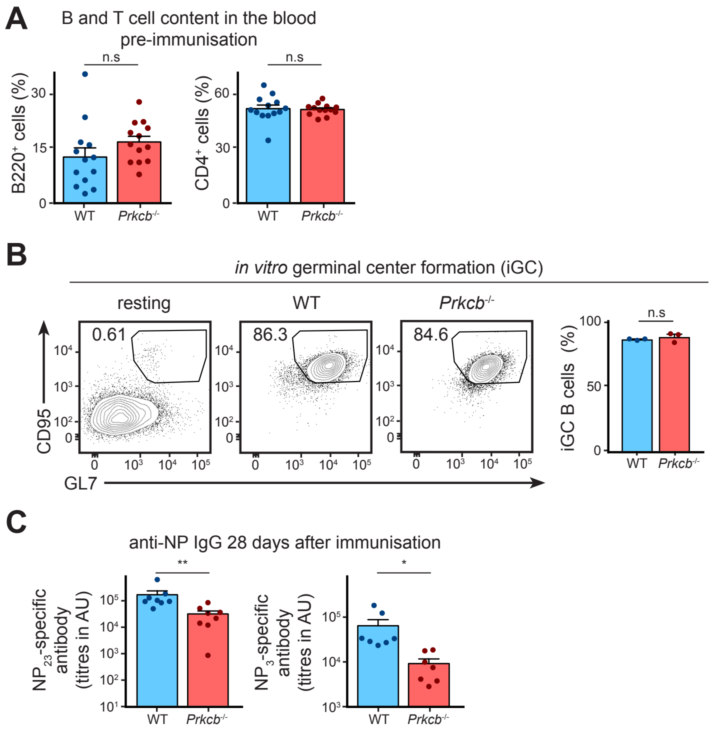

Figure S1. PKCβ depletion in the B cell compartment impairs T-dependent immune response. ***Related to Figure 1.***

(A) Quantifications showing percentages of B ($B220^+$) and $CD4^+$ T cells in the blood 8 weeks after bone marrow injection. Each dot represents one animal.

(B) Primary B cells from WT and *Prkcb*$^{-/-}$ mice cultured using iGC culture were analyzed using flow cytometry for the expression of GL-7 and CD95 after 4 days. Gated populations from three independent experiments were quantified.

(C) Sera were collected 28 days after NP-KLH immunization and anti-NP_{23} and anti-NP_3 IgG titres were measured using ELISA. All data are pooled from at least 2 independent experiments with at least 3 mice in each group. Error bars represent S.E.M.

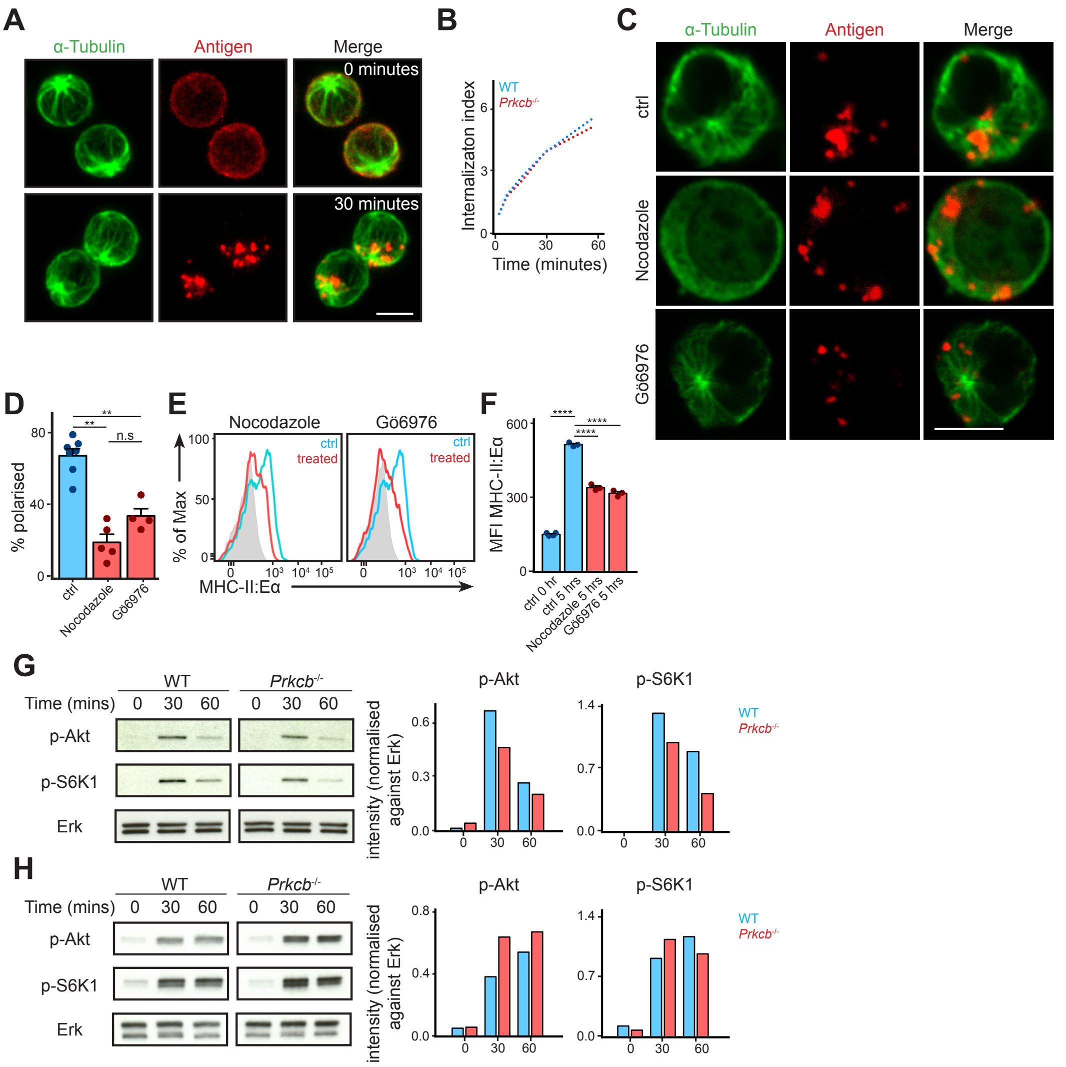

Figure S2. Antigen positioning is correlated with antigen presentation. ***Related to Figure 2.***

(A) Representative confocal images (63x objectives) showing primary B cells before and after stimulation with Alexa Fluoro 647-conjugated anti-IgM for 30 minutes. Bars, 5 µm. Data are representative of 2 independent experiments.

(B) Graph showing the internalization kinetics of antigen in WT and *Prkcb*$^{-/-}$ B cells stimulated (see *materials and* methods) for the indicated time points. Data are representative of 2 independent experiments.

(C) Representative confocal images (63x objectives) showing primary WT B cells stimulated with Alexa Fluoro 647-conjugated anti-IgM for 30 minutes in the absence and presence of microtubule depolymerizing agent nocodazole, PKC inhibitors Gö6976. Bars, 5 µm. Data are representative of 3 independent experiments.

(D) Samples were inspected using confocal microscopy and the proportions of cells with polarized antigen close to the MTOC were determined visually. Quantifications are pooled from 2 independent experiments with at least 40 cells per group and data analyzed using two-way ANOVA.

(E) Representative plots show the expression of surface MHC-II:Eα peptide after 5 hours detected by anti-MHC-II:Eα antibody of WT primary B cells that were incubated with microspheres in the presence of nocodazole or Gö6976. Data are representative of 2 independent experiments.

(F) MFI of MHC-II:Eα was quantified and analyzed using two-way ANOVA. Data are representative of 3 independent experiments.

(G and H) Western blots and the corresponding quantifications representing one experiment showing the induction of the PI3K pathway when B cells from WT and *Prkcb*$^{-/-}$ mice were given (G) coated-microspheres or (H) CpG and anti-IgM. Error bars represent S.E.M.

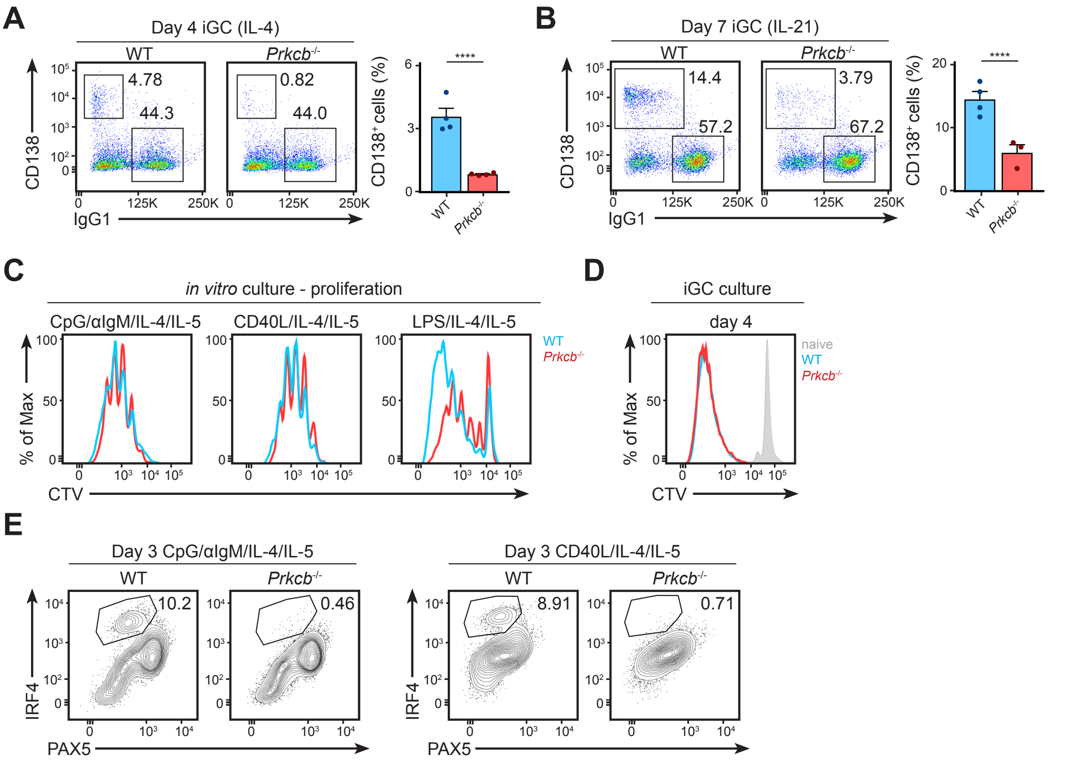

Figure S3. ***Prkcb$^{-/-}$*** **B cells are intrinsically defective in plasma cell differentiation.** ***Related to Figure 3.***

(A-B) Primary B cells from WT and *Prkcb$^{-/-}$* mice cultured using iGC culture were analyzed using flow cytometry for the expression of CD138 and IgG1 after (A) 4 days (IL-4 supplemented) and (B) 7 days (IL-4 and IL-21 supplemented) of culture. CD138^{hi} populations from three independent experiments were quantified.

(C) CTV-labelled primary B cells purified from WT and *Prkcb$^{-/-}$* mice were cultured for 4 days in the presence of CpG and anti-IgM, CD40L or LPS (all in the presence of IL-4 and IL-5). Representative histograms of CTV dilutions are shown.

(D) CTV-labelled primary B cells from WT and *Prkcb$^{-/-}$* mice cultured using iGC culture for 4 days. Representative histogram of CTV dilutions as analyzed using flow cytometry are shown.

(E) Primary B cells purified from WT and *Prkcb$^{-/-}$* mice were cultured with CpG and anti-IgM or CD40L in the presence of IL-4 and IL-5 for 3 days. Contour plots of the expressions of PAX5 and IRF4 on day 3 of culture with the specified stimuli combinations. All data are representative from at least 3 independent experiments. Error bars represent S.E.M.

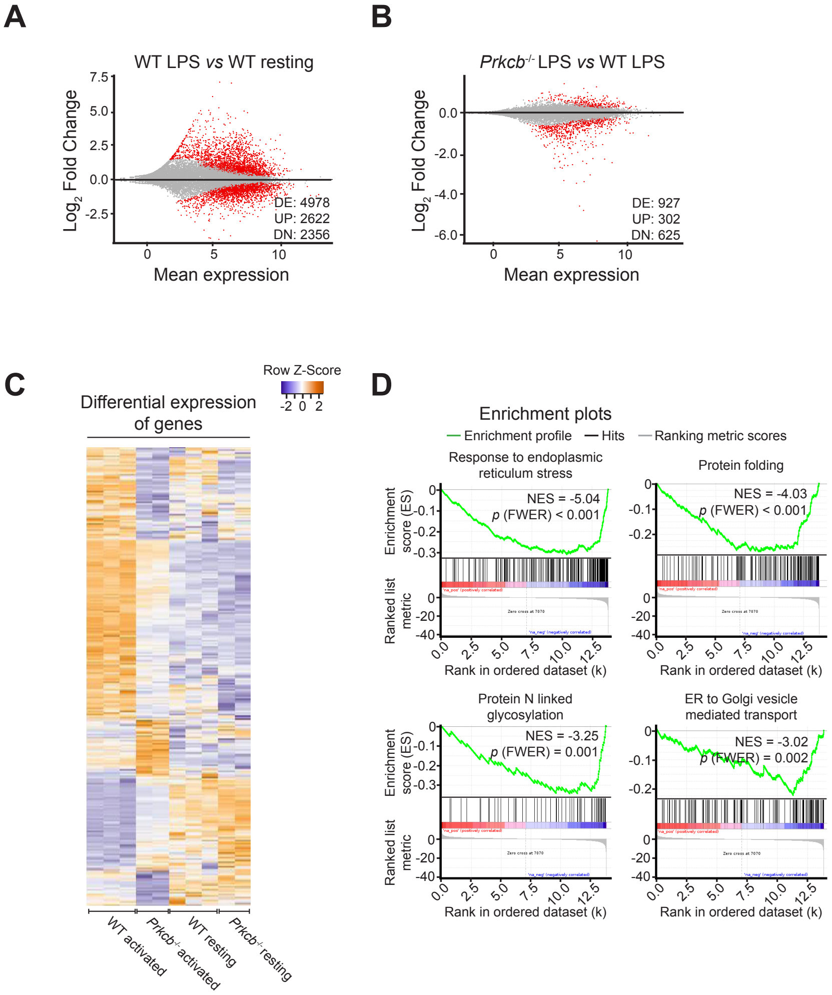

Figure S4. *Prkcb*$^{-/-}$ B cells expressed distinct transcriptome after activation compared to WT cells. *Related to Figure 3 and 4.*

(A and B) MA plots (log_2 Mean expression values on x-axis and log_2 Fold change on y-axis) showing in red differentially expressed genes (p-adj < 0.05) in (A) activated WT B cells compared to resting WT B cells and (B) activated *Prkcb*$^{-/-}$ B cells compared to activated WT B cells. DE: differentially expressed. UP: upregulated (log_2 fold change > 0). DN: down-regulated (log_2 fold change < 0).

(C) Hierarchical clustered heat map showing all 927 differentially expressed genes in B cells obtained from WT (n=3) and *Prkcb*$^{-/-}$ (n=2) mice before and after LPS stimulation *in vitro*.

(D) GSEA enrichment plots showing significantly underrepresented gene sets (within the top 58 hits) in activated *Prkcb*$^{-/-}$ B cells compared to activated WT cells.

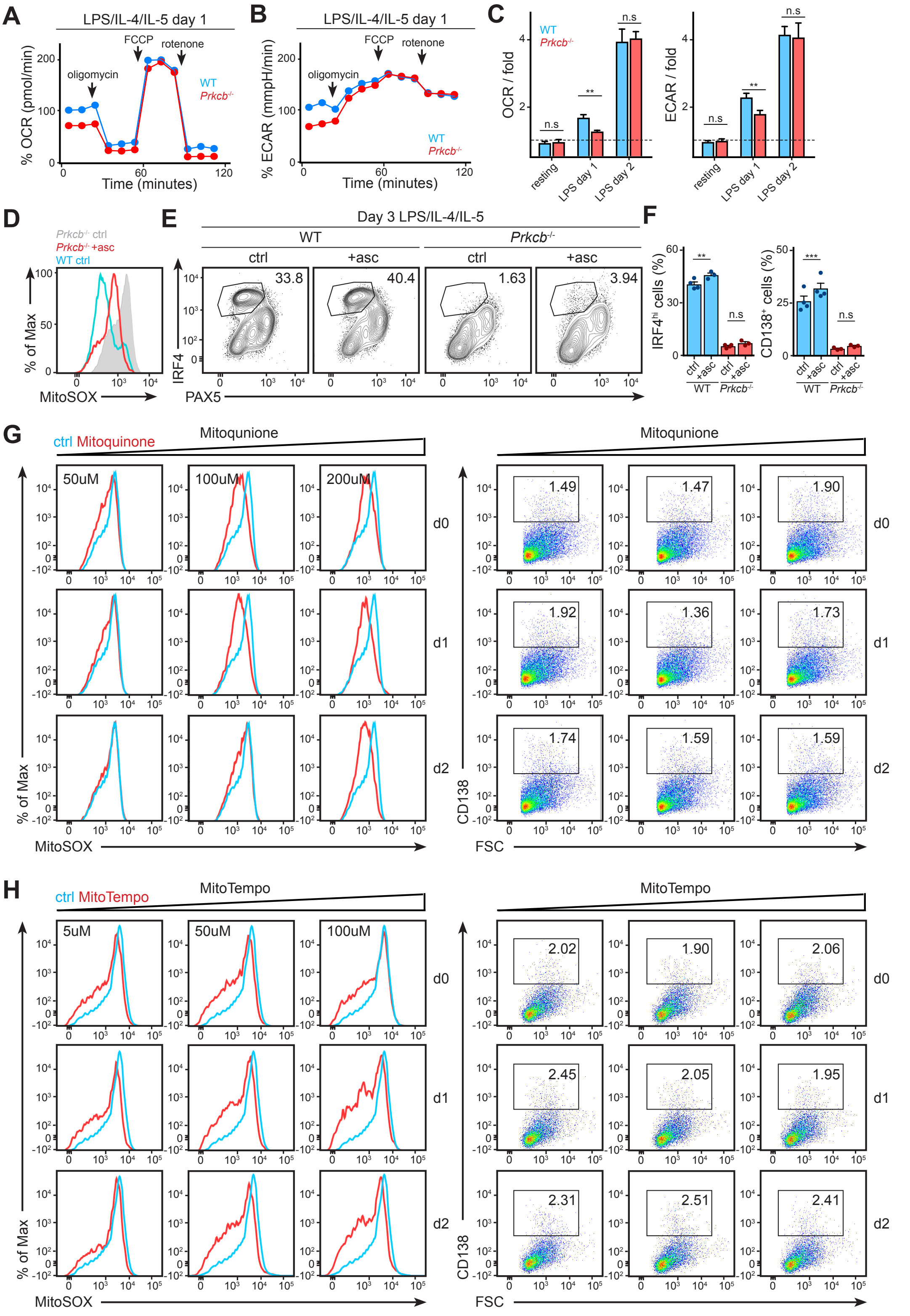

Figure S5. PKCβ functions as a regulator of mitochondrial homeostasis in activated B cells. ***Related to Figure 4.***

(A and B) Representative OCR (A) and ECAR (B) obtained from extracellular flux assay of WT and *Prkcb*$^{-/-}$ B cells stimulated with LPS, IL-4 and IL-5. Arrows indicate the time when each specified ETC modulators are added to the assay.

(C) Quantifications of oligomycin-sensitive OCR and steady state ECAR normalized to resting WT cells are shown. Data are pooled of at least 2 independent experiments with 2 mice in each group.

(D-F) Primary WT and *Prkcb*$^{-/-}$ B cells were cultured with LPS, IL-4 and IL-5 in the absence and presence of 200 µM ascorbic acid (added on day 1). (D) Accumulation of mROS and (E) contour plots of IRF4 and PAX5 expression. (F) Quantifications showing amount of IRF4^{hi} and CD138^{+} cells are shown in the same experimental setup. Data is representative of at least two independent experiments of two mice in each group.

(G and H) Primary WT and *Prkcb*$^{-/-}$ B cells were cultured with LPS, IL-4 and IL-5 in the absence and presence of (G) Mitoquinone or (H) MitoTempo. mROS accumulation and CD138 expression were analyzed using flow cytometry on day 3. Error bars represent S.E.M.

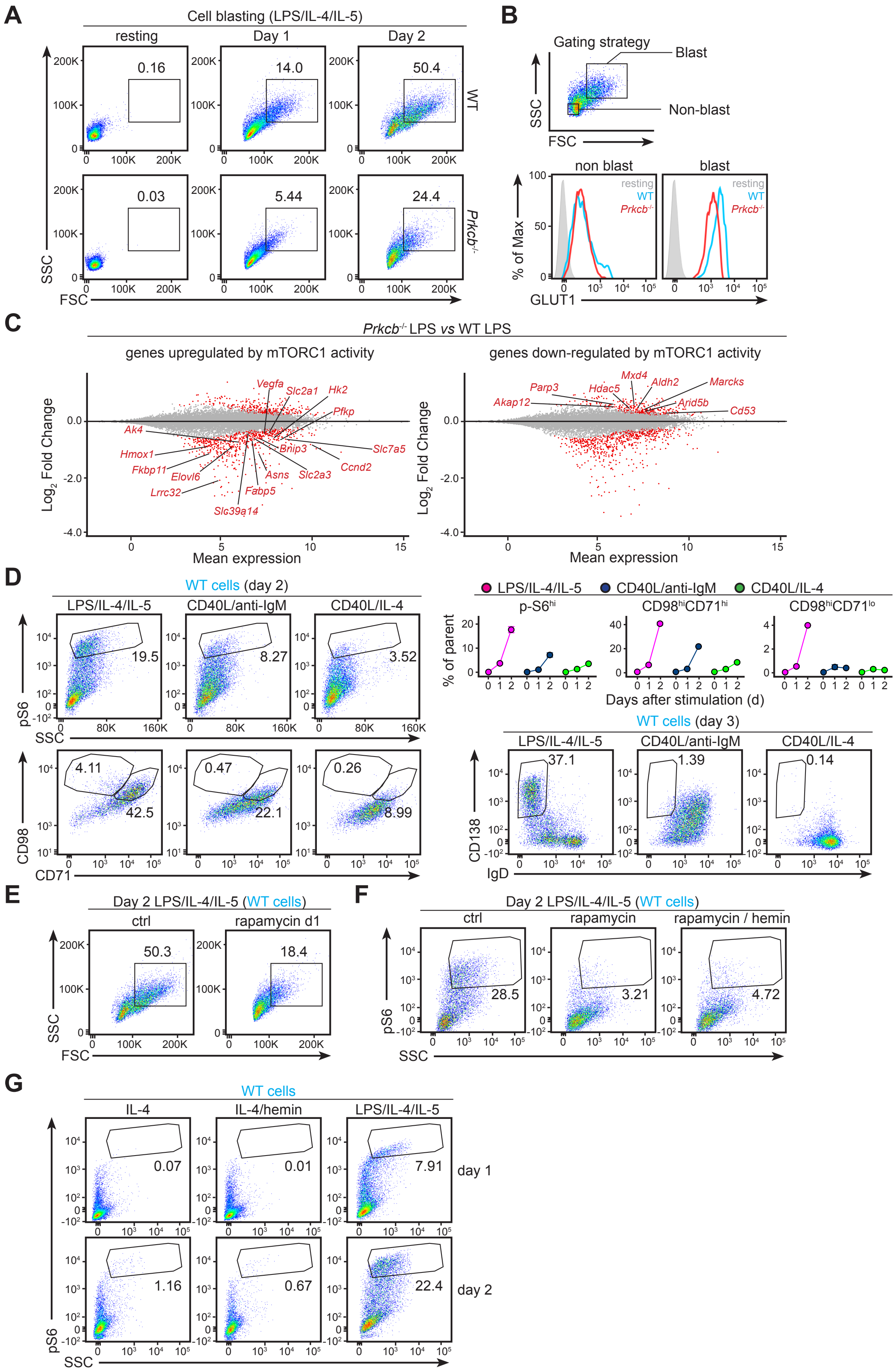

Figure S6. PKCβ regulates mTORC1 activity in activated B cells. ***Related to Figure 6.***

(A) Primary B cells purified from WT and *Prkcb*$^{-/-}$ mice were cultured with LPS, IL-4 and IL-5 for 2 days. The cell blasting kinetics was analyzed by flow cytometry on day 0, 1 and 2.

(B) B cells purified from WT and *Prkcb*$^{-/-}$ mice were cultured with LPS, IL-4 and IL-5 for 2 days. The cells were categorized into "blast" and "non-blast" using FSC. The total expression of GLUT1 within each population was measured using flow cytometry. GLUT1 expression in resting B cells is represented in grey.

(C) MA plots as in figure 3G. Highlighted in red are genes associated with (left) high mTORC1 activity and (right) low mTORC1 activity that are downregulated and upregulated, respectively, in activated *Prkcb*$^{-/-}$ cells compared to WT cells.

(D) Primary WT B cells were cultured in various stimuli as specified for 3 days. The mTORC1 activity (top left), CD98 and CD71 expression (bottom left) of the cells were monitored and quantitated (top right). The extent of plasma cell differentiation was measured on day 3 (bottom right).

(E) WT primary B cells were stimulated with LPS, IL-4 and IL-5 for 2 days. Rapamycin was added on day 1 of the culture and cell blasting was measured using flow cytometry on day 2.

(F) WT primary B cells were stimulated with LPS, IL-4 and IL-5 for 2 days. Rapamycin and hemin was added to the culture on day 1. Phosphorylation of S6 was measured using flow cytometry on day 2.

(G) WT primary B cells were cultured with IL-4 alone, IL-4 plus hemin and LPS, IL-4 and IL-5 for 2 days. Phosphorylation of S6 was analyzed using flow cytometry on day 1 and day 2. Error bars represent S.E.M.
